# Small molecule inhibition of voltage dependent anion channel 1 reroutes mitochondrial metabolite flux

**DOI:** 10.1016/j.mocell.2026.100369

**Published:** 2026-05-13

**Authors:** Seyed Majed Modaresi, Leilei Zhang, Amir Ata Saei, Morris Degen, Mohammad Khavani, Hassan Gharibi, Ákos Végvári, Zhiwei Ye, Jie Zhang, Evgeny Pavlov, Elizabeth A. Jonas, Kenneth D. Tew, Sebastian Hiller, Danyelle M. Townsend, Eduardo N. Maldonado

**Affiliations:** 1 Biozentrum, University of Basel, Basel, Switzerland; 2 Department of Pharmacology & Immunology, Medical University of South Carolina, Charleston, SC, USA; 3 Department of Microbiology, Tumor and Cell Biology, Karolinska Institute, Stockholm, Sweden; 4 Departments of Bioengineering and Mechanical Engineering, University of California, Berkeley, Berkeley, CA, USA; 5 Division of Chemistry I, Department of Medical Biochemistry and Biophysics, Karolinska Institutet, Stockholm, Sweden; 6 Department of Molecular Pathobiology, New York University, New York, NY, USA; 7 Department of Internal Medicine (Endocrinology) and Neuroscience, Yale University, New Haven, CT, USA; 8 Department of Drug Discovery & Biomedical Sciences Medical University of South Carolina, Charleston, SC, USA; 9 Hollings Cancer Center, Medical University of South Carolina, Charleston, SC, USA

**Keywords:** Cancer metabolism, Glycolysis, Mitochondria, SC18, VDAC1

## Abstract

Voltage dependent anion channels (VDACs 1, 2 and 3) in the outer mitochondrial membrane control the flux of anions and oxidizable substrates that sustain mitochondrial metabolism. Nicotinamide adenine dinucleotide (NADH) closes VDAC by binding to a pocket, conserved in all isoforms, located in the inner wall of the channel. Previously, we identified the small molecule SC18 that targets the NADH-binding pocket of VDAC1 employing computational analysis. Here, we explored the interaction between SC18 and VDAC1 using high-resolution nuclear magnetic resonance spectroscopy and molecular dynamics simulations. Atomically resolved data precisely confirmed the computational results, showing that SC18 binds to a site on VDAC1 that partially overlaps with the NADH binding pocket. SC18, in the presence of NADH blocked the conductance of VDAC1 reconstituted in lipid bilayers. To determine the metabolic effect of SC18, we combined readouts of mitochondrial metabolism and glycolysis with functional metabolomics and proteomics. Short-term treatment with SC18 inhibited mitochondrial metabolism and adenosine triphosphate production. Treatment over 24 h and 48 h further reduced mitochondrial uptake of pyruvate and glutamine, utilization of tricarboxylic acid cycle intermediates, as well as lipid, DNA and amino acid synthesis. Concomitant with the inhibition of mitochondrial metabolism, cellular uptake of glucose and glutamine increased in parallel with augmented lactate release. These results indicate that compensatory enhanced glycolysis sustains adenosine triphosphate production after impaired mitochondrial function induced by SC18 blockage of VDAC1. Our work sets a mechanistic foundation for VDAC1 inhibition as a novel strategy to target and reprogram cancer metabolism through modulation of the biosynthetic ability of mitochondria.

## Graphical Abstract

**Figure:**
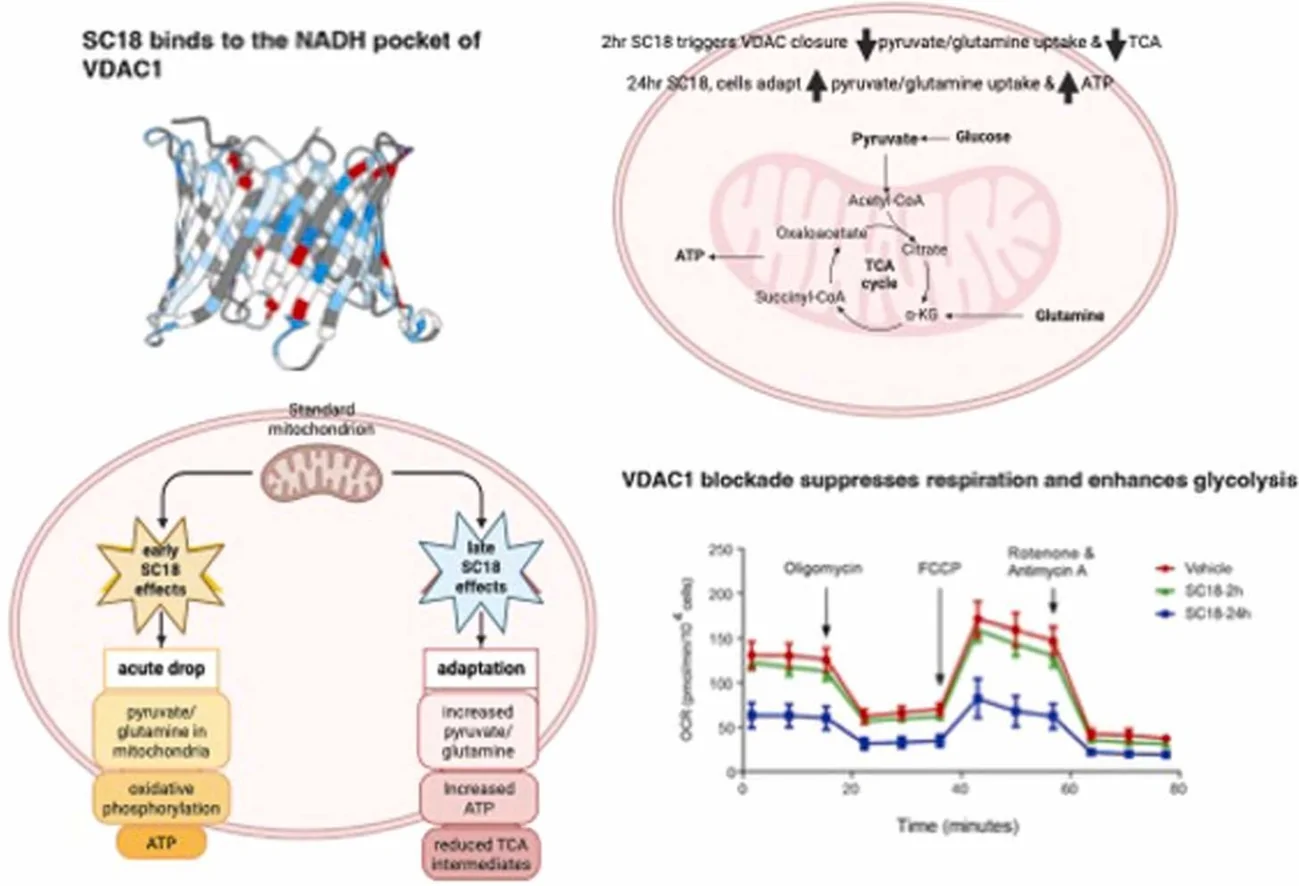

## INTRODUCTION

Mitochondria contribute to cancer cell proliferation by producing adenosine triphosphate (ATP) and supplying metabolic intermediates essential for synthesis of macromolecules used to generate biomass. Maintenance of mitochondrial metabolism in cancer cells depends on an adequate influx of oxidizable substrates, ADP, inorganic phosphate, and small ions into mitochondria, as well as the efflux of ATP and metabolic intermediates to the cytosol.

To reach the mitochondrial matrix, most anionic metabolites and small ions cross the outer mitochondrial membrane (OMM) through voltage-dependent anion channels (VDACs) 1, 2 and 3 (Colombini, 2012, Colombini and Blachly-Dyson, 1996, Rostovtseva and Colombini, 1997). In most mammalian cells, VDAC1 and 2 are the predominant isoforms, whereas VDAC3 is the least expressed, except for testis and spermatozoa (Colombini and Blachly-Dyson, 1996, De Pinto et al., 2010). Overall, VDAC (∼30 kDa) operates as a biological switch that maximizes the flux of metabolites for optimal mitochondrial function in the open state, whereas during the closed state, it lowers mitochondrial metabolism (Fang and Maldonado, 2018, Maldonado, 2017, Rovini et al., 2022). Regulation of this channel has an amplifying effect on metabolism and bioenergetics (Colombini, 2004, Maldonado, 2017, Rostovtseva and Colombini, 1997). Moreover, the magnitude and duration of VDAC opening may serve as an adjustable rheostat with a range of operational levels (Heslop et al., 2021). Because of this functional role, we have proposed VDAC as a pharmacological target to induce mitochondrial dysfunction and cancer cell death (Fang and Maldonado, 2018, Heslop et al., 2021, Rovini et al., 2022).

Although nicotinamide adenine dinucleotide (NADH) has long been known to induce VDAC closure (Zizi et al., 1994), the binding site responsible was identified only recently. β-NADH binding to a NADH-binding pocket in VDAC1, which amino acid sequence is conserved in all VDAC isoforms, reduces VDAC conductance by steric occlusion of the pore without conformational changes at the α-helix domain (Bohm et al., 2020). Using computational analysis combined with biological assays, we recently identified SC18 as a small-molecule inhibitor targeting the NADH-binding pocket of VDAC1. Short-term treatment with SC18, decreased mitochondrial membrane potential (ΔΨm), mitochondrial NADH and cellular ATP levels (Heslop et al., 2022). We also demonstrated that SC18 not only kills hepatocarcinoma cells as standalone treatment but synergizes with the FDA-approved hepatocellular carcinoma (HCC) drugs-sorafenib, regorafenib, and lenvatinib (Ventura et al., 2023). Targeting VDAC1 could thus exploit a metabolic vulnerability in cancer, potentially complementing existing therapies. However, the precise molecular mechanisms by which SC18 exerts its pharmacological effects remained poorly understood.

In this study, we aimed to confirm the binding site and the effects of SC18 on VDAC conductance, mitochondrial metabolism and cellular metabolism. We used a multidisciplinary approach combining solution nuclear magnetic resonance (NMR) spectroscopy, AlphaFold 3 co-folding, molecular dynamics simulations in explicit lipid bilayers, electrophysiology, functional metabolomics, quantitative proteomics, and classical readouts of mitochondrial function. We show that SC18 binds to a site on VDAC1 that partially overlaps with the NADH binding pocket, cooperatively reducing VDAC conductance in the presence of NADH. This reduction in conductance restricts the mitochondrial uptake of pyruvate, acyl carnitines and metabolic intermediaries decreasing mitochondrial metabolism. In parallel, there is a compensatory increase in glycolysis to sustain ATP production. SC18 treatment also induces downregulation of aminoacyl-tRNA synthetases, and enzymes involved in nucleotide and lipid biosynthesis, which is consistent with compromised anabolic capacity downstream of mitochondrial substrate restriction. Overall, our findings establish a mechanistic foundation for VDAC1 inhibition as a novel strategy to target cancer metabolism.

## RESULTS

### SC18 Binds to Voltage Dependent Anion Channel 1 in a Partially Shared Site With the NADH Binding Pocket

To identify the SC18 binding site on VDAC1, we performed molecular docking simulations using a previously described VDAC1(PDB:6TIR)-SC18 docking model to refine the model and resolve structural clashes (Bohm et al., 2020, Ventura et al., 2023). This was followed by 200 ns molecular dynamics (MD) simulations in triplicates to examine the dynamic behavior of the complexes and assessment of the stability of VDAC1 complexes with SC18 and NADH (Fig. 1A, B). Both SC18 and NADH formed stable complexes with VDAC1, remaining within the channel’s pocket as evidenced by converged RMSD trajectories (Fig. 1A). To further investigate the relative stabilities of VDAC1-ligand complexes and obtain qualitative trends in binding energetics, complexation energies were estimated using the MM-GBSA method. It should be noted that MM-GBSA provides semi-quantitative estimates and the values reported here should be interpreted as qualitative indicators of relative binding favorability rather than absolute binding free energies. The calculated Gibbs binding energy estimates (ΔG_tot_) confirmed the stability and favorability of the complexes. Qualitative assessment of the MD data suggested that the ΔG_tot_ values for the SC18 and NADH complexes with VDAC1 were −22.10 ± 1.96 and −43.10 ± 1.70 kcal/mol, respectively; these estimates qualitatively supported that SC18 occupies a stable position within the channel pocket. To experimentally assess the binding site, we prepared [^15^N,^1^H] isotopically spin-labeled human VDAC1 and conducted a titration followed by Transverse Relaxation Optimized Spectroscopy (TROSY) NMR experiments with 3 mM and 6 mM of SC18 (Supp. Figs. 1 and 2). Due to the hydrophobic nature of SC18, SC18 was dissolved in DMSO-d6 and titrated into the NMR sample tube as described in Methods. A matched volume of DMSO-d6 in the same buffer served as vehicle control. All 2D TROSY experiments were performed under LDAO micelle conditions where resonance assignment coverage is the highest. Comparing amide resonance peak intensities and chemical shifts enabled identification of the SC18 binding site on VDAC1 at the atomic level (Fig. 1C-E).

**Fig. 1:**
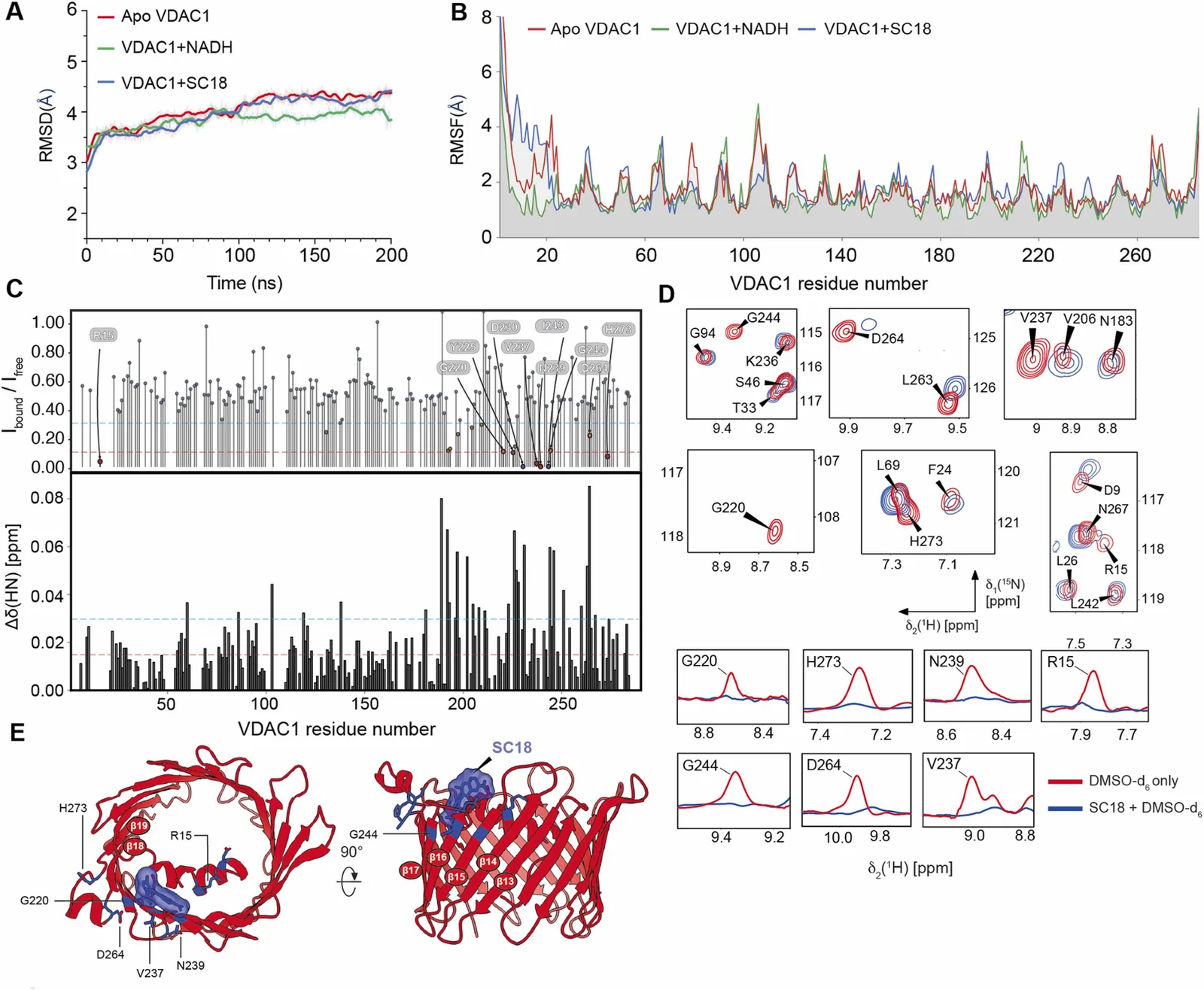
Structural analysis of SC18 binding to VDAC1. (A) Backbone root-mean-square deviation (RMSD) of VDAC1 over simulation time in the apo state (red), in complex with NADH (green), and with SC18 (blue), indicating stable trajectories with modest ligand-dependent differences. The simulations were done for 200 ns in three replicates. (B) Residue-wise root-mean-square fluctuation (RMSF) of VDAC1 for apo (red), NADH-bound (green), and SC18-bound (blue) conditions, highlighting localized changes in flexibility upon ligand binding. (C) Top: Ratio of bound to free peak intensities (I_bound_/I_free_) from two-dimensional TROSY NMR measurements mapped across assigned VDAC1 residues reconstituted in LDAO micelles, identifying residues affected by SC18 interaction; Residues cluster along β-strands 13–17, suggesting a putative SC18 binding site on VDAC1. Bottom: Corresponding amide chemical shift perturbations (ΔδHN, ppm), indicating residues experiencing significant environmental changes upon ligand binding, which converge on the same β-strand region. The red and blue dashed lines indicate threshold levels used to define significant decreases in peak intensity (top) and significant chemical shift perturbations (bottom), respectively. (D) Representative regions of 2D TROSY NMR spectra showing residue-specific chemical shift changes and/or intensity alterations upon SC18 binding (blue) compared to control (red), with selected residues annotated. One-dimensional cross-sections for representative residues are shown below, illustrating signal intensity attenuation upon interaction with SC18. (E) Structural mapping of NMR-identified residues affected by SC18 binding onto the VDAC1 structure. Residues with significant perturbations cluster in defined regions of the β-barrel, suggesting a localized binding interface. SC18 is shown as a blue surface representation. All Two-dimensional NMR data were recorded for human VDAC1 reconstituted in LDAO micelles. Abbreviations: NMR: nuclear magnetic resonance.

Notably, most of the amide resonances of VDAC1 that exhibited line broadening in the TROSY spectra were concentrated near the NADH-binding pocket, located at the interface between the N-terminal helix and the interior wall of the VDAC1 β-barrel (Fig. 1E, Supp. Fig. 3). Pronounced chemical shift perturbations (CSPs) were observed in β-strands 13–18 and their corresponding connecting loops, further supporting the SC18 binding site on VDAC1 (Fig. 1C). Collectively, residues implicated in binding, as indicated by significant NMR signal attenuation (I_bound_/I_free_ < 0.1) and chemical shift perturbation, include R15 on the N-terminal helix, as well as G244, V237, G220, D264, H273, and N239 within the β-barrel structure (Fig. 1C-E). Among these, five residues showed complete or near-complete signal disappearance (I_bound_/I_free_ < 0.1); additional residues displayed moderate attenuation (I_bound_/I_free_ = 0.10–0.21), (Fig. 1C; Table 1). Although the N-terminal helix exhibited conformational flexibility across the MD ensemble regardless of ligand, SC18 did not induce significant chemical shift perturbations in this region, suggesting that SC18 binding does not cause substantial repositioning of the α-helical domain under our experimental conditions (Fig. 1B, C). Together, NMR and molecular dynamics analyses demonstrate that SC18 binds stably and favorably to VDAC1 at a site that partially overlaps with the β-NADH (hereafter called NADH) binding pocket.

**Table 1:** NMR ¹H–¹⁵N TROSY-HSQC peak intensity ratios (I_bound_/I_free_) for residues displaying signal attenuation upon SC18 binding to ¹⁵N-labeled hVDAC1 E73V in LDAO micelles

Residue	δ¹H_free (ppm)	δ¹⁵N_free (ppm)	CSP(ppm)	I_free (a.u.)	I_bound (a.u.)	I_bound/I_free	Signal change
N239	—	—	—	228,402	0	0.000	Disappeared
V237	9.008	117.38	0.003	485,382	10,247	0.021	Severe (>90%)
R15	7.377	117.86	0.013	159,817	5470	0.034	Severe (>90%)
H273	7.245	120.77	0.013	427,730	29,589	0.069	Severe (>90%)
G220	8.617	108.25	0.055	183,752	19,111	0.104	Significant (80–90%)
G244	9.343	114.91	0.060	252,727	27,840	0.110	Significant (80–90%)
D264	9.914	124.85	0.085	249,412	53,511	0.214	Moderate (70–80%)

NMR: nuclear magnetic resonance.

### SC18 and NADH co-binding reduces voltage dependent anion channel 1 conductance

To provide an independent structural perspective on the SC18 binding mode and evaluate isoform selectivity, we employed AlphaFold 3 (AF3) co-folding of VDAC1 with SC18. The resulting model yielded high-confidence metrics (ipTM = 0.94, minimum predicted aligned error PAEₘᵢₙ ≈ 1.02 Å), confirming a stable and well-defined complex. In the AF3 model, SC18 occupies the NADH-binding pocket at the interface of the N-terminal helix and β-strands 17–19, fully consistent with the ^2^D NMR data (Fig. 2A-C). To assess whether this binding site is conserved, we performed AF3 co-folding with VDAC2 and VDAC3 under identical conditions. All three isoforms accommodated SC18 in a structurally homologous binding mode (Fig. 2A, C and Table 2), consistent with the high sequence conservation of the NADH-binding pocket across VDAC isoforms. These findings support the fact that SC18 is a pan-VDAC inhibitor and provide an atomically resolved model that integrates well with the experimental NMR binding map.

**Fig. 2:**
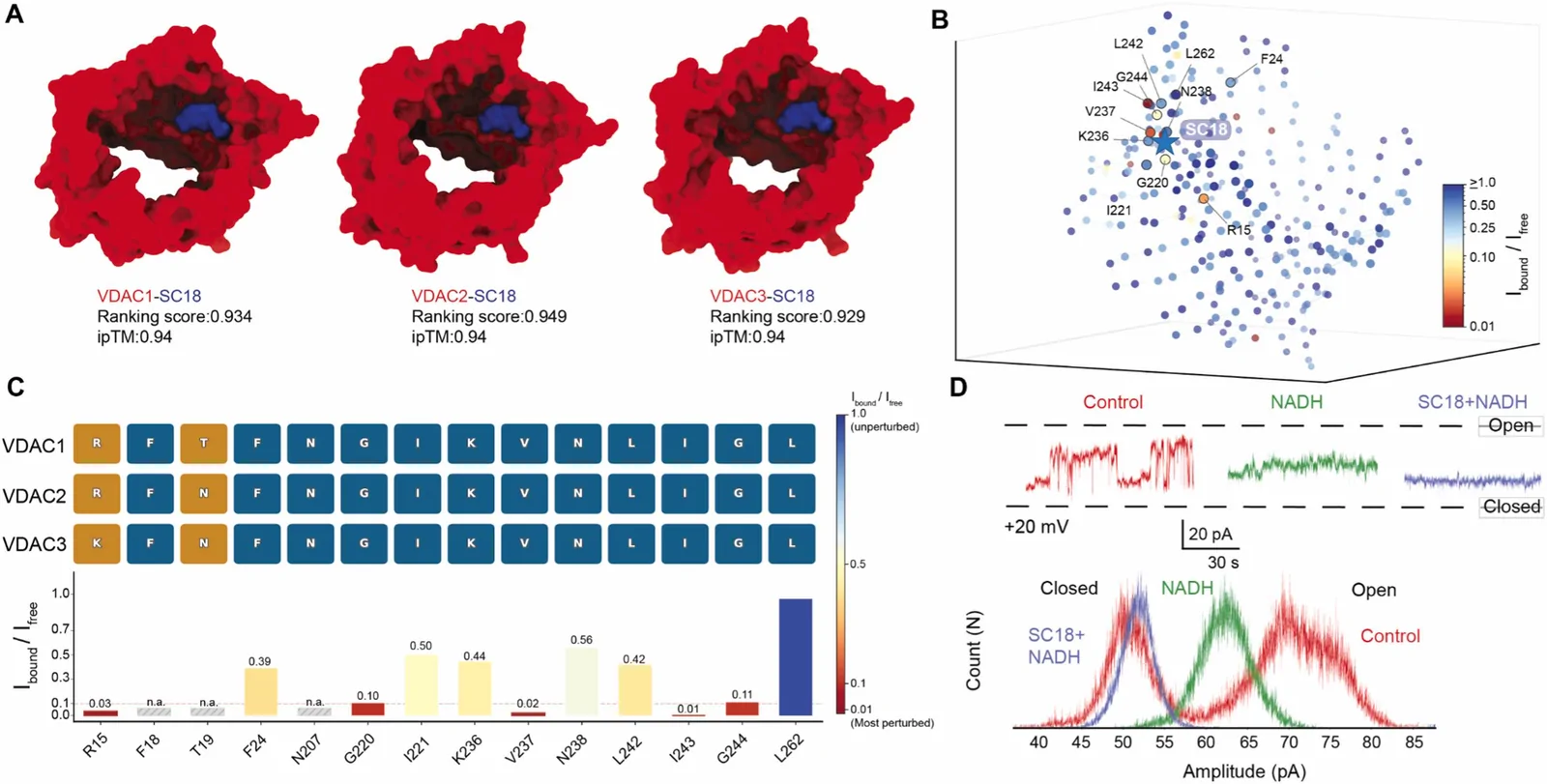
SC18 engages a conserved intra-barrel binding site in VDAC and stabilizes the closed channel state. (A) AlphaFold3-predicted binding poses of SC18 (blue) within the lumen of VDAC1, VDAC2, and VDAC3 (red surfaces), viewed from the cytoplasmic face. All three isoforms yield high-confidence predictions (ipTM = 0.94; Ranking scores 0.929–0.949), indicating a consistent binding geometry across the VDAC isoforms. (B) NMR signal intensity perturbation (I_bound_/I_free_) mapped onto the Cα backbone of VDAC1 arranged in native barrel topology. Each sphere represents one residue; color encodes signal attenuation upon SC18 addition on a log scale from unperturbed (blue, I_bound_/I_free_ ≥ 1.0) to strongly broadened (red, I_bound_/I_free_ = 0.01). The SC18 binding centroid (blue star) localizes to the upper inner wall of the barrel. Residues forming the binding epitope (as quantified in panel C) are labeled; the cluster of perturbed residues at β-strands 13–17 (V237, I243, G244, L242, N238, K236) and the N-terminal helix anchor R15 define the SC18 footprint. (C) Conservation of the SC18 binding epitope across VDAC isoforms. (Top) Heatmap of equivalent pore-lining residues in VDAC1, VDAC2, and VDAC3 colored by I_bound_/I_free_, demonstrating that the binding pocket geometry is conserved despite sequence variation at positions R/K15 (both positively charged), residues lacking an assigned cross-peak in the bound state are marked n.a. (Bottom) Quantitative I_bound_/I_free_ values for each epitope residue. Residues with values near zero (R15 = 0.03, V237 = 0.02, I243 = 0.01) indicate near-complete signal loss consistent with direct contact or immobilization by SC18, whereas residues such as I221, K236, and L242 show intermediate attenuation (0.42–0.50) reflecting second-shell effects. L262 remains essentially unperturbed (0.96), serving as an internal negative control. (D) Single channel recording of VDAC1 reconstituted into lipid bilayers at a holding voltage of +20 mV where the panel shows representative traces of channel activity following reagent addition, with peak open and closed states indicated. Bottom panel shows amplitude histograms, representing the distribution of recorded data points at various amplitudes (x-axis). Each histogram, color-coded for control, NADH, and NADH+SC18, highlights the mean amplitude for the corresponding trace. The control histogram reveals the open and closed states of the channel, whereas the addition of NADH followed by SC18 induces a sub-conductance state in VDAC1. Abbreviations: NADH, nicotinamide adenine dinucleotide; NMR: nuclear magnetic resonance.

**Table 2:** AlphaFold3 prediction confidence metrics for SC18 (lig1) and NADH (lig2) docked to VDAC Isoforms

Protein	Ligands	PAE VDAC–SC18	PAE VDAC–β-NADH	PAE VDAC–SC18-β-NADH	pTM VDAC–SC18	pTM VDAC–β-NADH	pTM VDAC–SC18-β-NADH	Ranking	ipTM	pTM
VDAC2	lig1	1.08	—	—	0.94	—	—	0.95	0.94	0.87
VDAC1	lig1	1.02	—	—	0.94	—	—	0.93	0.94	0.91
VDAC3	lig1	1.04	—	—	0.94	—	—	0.93	0.94	0.88
VDAC3	lig1 +lig2	1.05	1.94	3.42	0.95	0.84	0.47	0.88	0.88	0.89
VDAC2	lig1 + lig2	1.23	3.21	4.63	0.94	0.76	0.44	0.86	0.83	0.87
VDAC3	lig2	2.45	—	—	0.80	—	—	0.81	0.80	0.83
VDAC1	lig1 + lig2	1.04	3.95	5.43	0.94	0.67	0.31	0.80	0.78	0.89
VDAC1	lig2	3.01	—	—	0.71	—	—	0.74	0.71	0.87
VDAC2	lig2	3.59	—	—	0.69	—	—	0.73	0.69	0.82

NADH, nicotinamide adenine dinucleotide; PAE, predicted aligned error (Å; lower values indicate higher inter-chain confidence); pTM, predicted template-modeling score; ipTM, interface pTM score (higher values indicate higher confidence in the modeled interface). Proteins and ligand combinations tested are indicated in the first two columns. Ranking reflects the AlphaFold3 model seed ranking.

—: metric not applicable for the given ligand combination.

To experimentally assess SC18 binding under physiologically relevant conditions, we employed Saturation Transfer Difference (STD) NMR spectroscopy comparing SC18 interaction with VDAC1 reconstituted in lipid nanodiscs versus LDAO detergent micelles. Notably, cryo-EM structure determination of VDAC1 in the same membrane scaffold protein 1D1 (MSP1D1) nanodisc system confirmed that the protein adopts a well-preserved native conformation in this environment (Supp. Figs. 4, 5). At a 1:100 VDAC1:SC18 ratio, in the SC18-specific aromatic region, a clear STD signal was detected only when VDAC1 was present in lipid nanodiscs, while the micelle condition yielded no resolvable signal above background (Supp. Fig. 6). This is consistent with hydrophobic sequestration of SC18 by a detergent suppressing productive engagement with the channel and explains the high ligand concentration needed for TROSY experiments. Together, these data establish that SC18 binding to VDAC1 is favored in a lipid bilayer context.

To functionally assess the structural and computational findings, we conducted single-channel electrophysiological measurements of VDAC1 reconstituted in planar lipid bilayers. VDAC1 remained mostly in the fully open state at voltages between +40 and –40 mV and transitioned to steady sub-conductance states at voltages above +40 mV or below –40 mV. To test the effects of ligands, the channel was maintained at either +20 or +40 mV for several minutes to record baseline activity and ensure the absence of spontaneous transitions to sub-conductance states. Upon NADH addition, the channel shifted to a sub-conductance state, showing a moderate reduction in amplitude, consistent with previous findings (Bohm et al., 2020). When SC18 was subsequently introduced to NADH-treated channel, the current amplitude was further reduced, resulting in a low-conductance state centered around ∼50 pA (Fig. 2D). The additional suppression of conductance therefore indicates that NADH and SC18 together stabilize a low-conductance state of VDAC1. We next examined ligand-induced changes in channel architecture by analyzing the pore geometry along the VDAC1 axis using MD trajectories. In the apo state, the channel displayed a relatively wide and solvent-accessible pore (minimum radius ∼5.0 Å) without pronounced constrictions, consistent with the open, conductive state of VDAC1. Binding of either NADH or SC18 alone led to a localized narrowing of the pore within the membrane-spanning region, indicating partial obstruction of the ion-conducting pathway. Notably, the co-bound SC18–NADH state produced the most pronounced effect, reducing the minimum pore radius to ∼3.8 Å and generating a sharper constriction profile along the channel axis.

### Voltage Dependent Anion Channel 1 Inhibition by SC18 Triggers Proteome Remodeling Affecting Lipid, Nucleotide and Amino Acid Synthesis

To determine whether SC18 alters the mitochondrial and cellular proteome in response to VDAC1 inhibition, we treated Huh7 human hepatocellular carcinoma cells with SC18 (30 µM) for 24 h and 48 h and performed quantitative proteomics.

Volcano plots showed upregulation of all three VDAC isoforms in a time-dependent manner following SC18 treatment (Fig. 3A-C). This pattern is consistent with compensatory feedback regulation of drug targets, a phenomenon previously reported for other anticancer compounds (Mitchell et al., 2023, Saei and Beusch, 2019, Saei et al., 2018). These findings suggest that cells attempt to compensate for VDAC inhibition by increasing channel abundance.

**Fig. 3:**
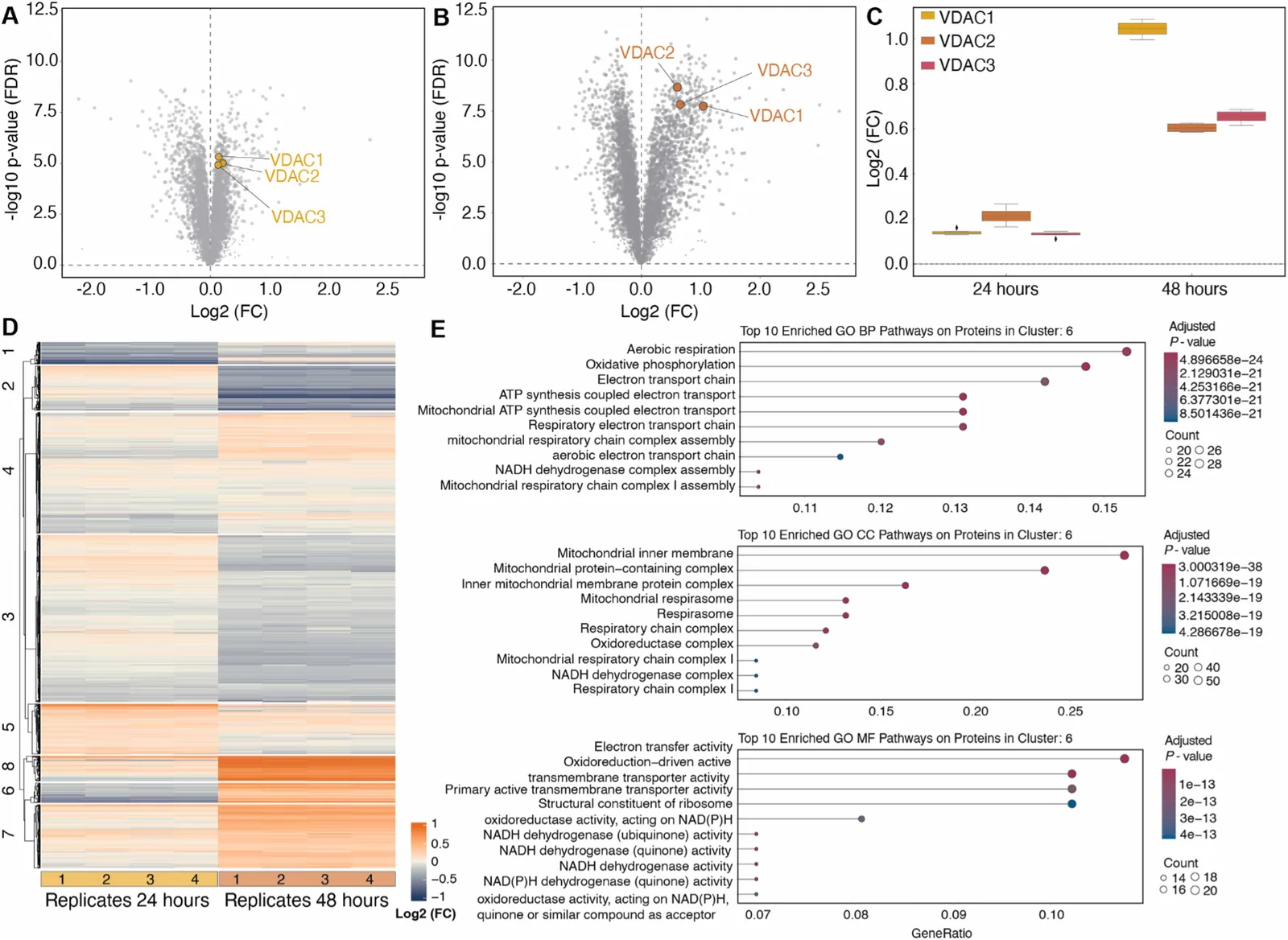
Proteome-wide analysis of the effect of SC18 on liver cancer cells. (A) Volcano plot showing differential protein expression in Huh7 cells treated with SC18 (30 µM) for 24 h and (B) 48 h where all three VDAC isoforms are upregulated. (C) Box plots representing the relative expression ratios of VDAC1, VDAC2, and VDAC3 after 24 and 48 h of SC18 treatment. VDAC1 upregulation at 48 h is statistically significant (p = 0.0024). (D) Heatmap of log2 fold-changes in protein abundance across biological replicates at 24 and 48 h, revealing time-dependent proteomic remodeling. (E) Gene Ontology (GO) enrichment analysis of proteins in Cluster 6 (from panel D) showing significant enrichment of pathways related to oxidative phosphorylation, mitochondrial respiratory chain complexes, and oxidoreductase activity, indicating mitochondrial stress responses following VDAC1 inhibition.

Hierarchical clustering of the proteomic data revealed clear temporal patterns, with more pronounced changes in protein expression occurring at 48 h (Fig. 3D). Specifically, Cluster 6 showed a strong upregulation of proteins involved in mitochondrial function, which is consistent with the increase in VDAC expression. Gene Ontology (GO) enrichment analysis of this cluster (Fig. 3E) identified cellular components associated with the mitochondrial inner membrane, respiratory chain complexes, and the mitochondrial respirasome, all of them related to oxidative phosphorylation and mitochondrial ATP synthesis (Fig. 3E). Furthermore, molecular function analysis revealed overrepresentation of oxidoreductase and NAD(P)H dehydrogenase activities, consistent with VDAC1’s known involvement in redox balance and metabolite exchange (Colombini, 2012, Fang and Maldonado, 2018, Junco et al., 2024). These enriched pathways reflect both a mitochondrial response and compensatory adjustments after impaired mitochondrial function induced by VDAC1 inhibition. Beyond the mitochondrial signature, proteomic analysis revealed a coordinated downregulation of proteins related to the synthesis of lipids and DNA.

SCD (Stearoyl-CoA desaturase), FADS1 and 2 (Fatty acid desaturases 1 and 2), ELOVL5 (ELOVL fatty acid elongase 5), ACSS2 (Acyl-CoA synthetase short chain family member 2), ACSL3 (Acyl-CoA synthetase long chain family member 3), and FASN (Fatty acid synthase) were downregulated both at 24 h and 48 h. Decreased expression of these enzymes, involved in fatty acid elongation and desaturation, disrupts the synthesis of several lipids, such as phospholipid, triglycerides and cholesterol esters.

PCLAF (PCNA Clamp-Associated Factor), RRM1 (ribonucleotide reductase catalytic subunit M1), RRM2 ribonucleotide reductase regulatory subunit M2, TYMS (thymidylate synthetase), PCNA (proliferating cell nuclear antigen), DHFR (Dihydrofolate reductase), Thymidine kinase 1 (TK1), were downregulated at 24 h. All these enzymes are involved in nucleotide and DNA synthesis and repair. Interestingly, RRM2, PCLAF, and DHFR were upregulated at 48 h after treatment with SC18, suggesting that the suppression of nucleotide synthesis might be transient.

SC18 treatment also downregulated 7 aminoacyl-tRNA synthetases (AARS1, EPRS1, GARS1, LARS1, MARS1, SARS1, VARS1) and translation initiation factors EIF4B (Eukaryotic translation initiation factor 4B) and EIF2A (Eukaryotic translation initiation factor 2A). Aminoacyl-tRNA synthetases key enzymes that catalyze the binding of specific amino acids to their corresponding tRNA molecules. Taken together, downregulation of all these proteins indicated impaired protein biosynthesis 48 h after treatment.

Taking together the proteomic data and Gene Ontology enrichment indicates that after VDAC1 inhibition, there is a compensatory increase in the expression of VDACs, and other mitochondrial proteins related to oxidative phosphorylation. In parallel, there is downregulation of enzymes related to lipid, nucleotide and amino acid synthesis consistent with the impaired exchange of metabolic intermediaries between mitochondria and cytosol caused by VDAC1 inhibition (Fig. 6). These findings support a model where SC18 treatment impairs not only mitochondrial bioenergetic function but also the cellular capacity to sustain protein, lipid, and nucleotide biosynthesis

**Fig. 4:**
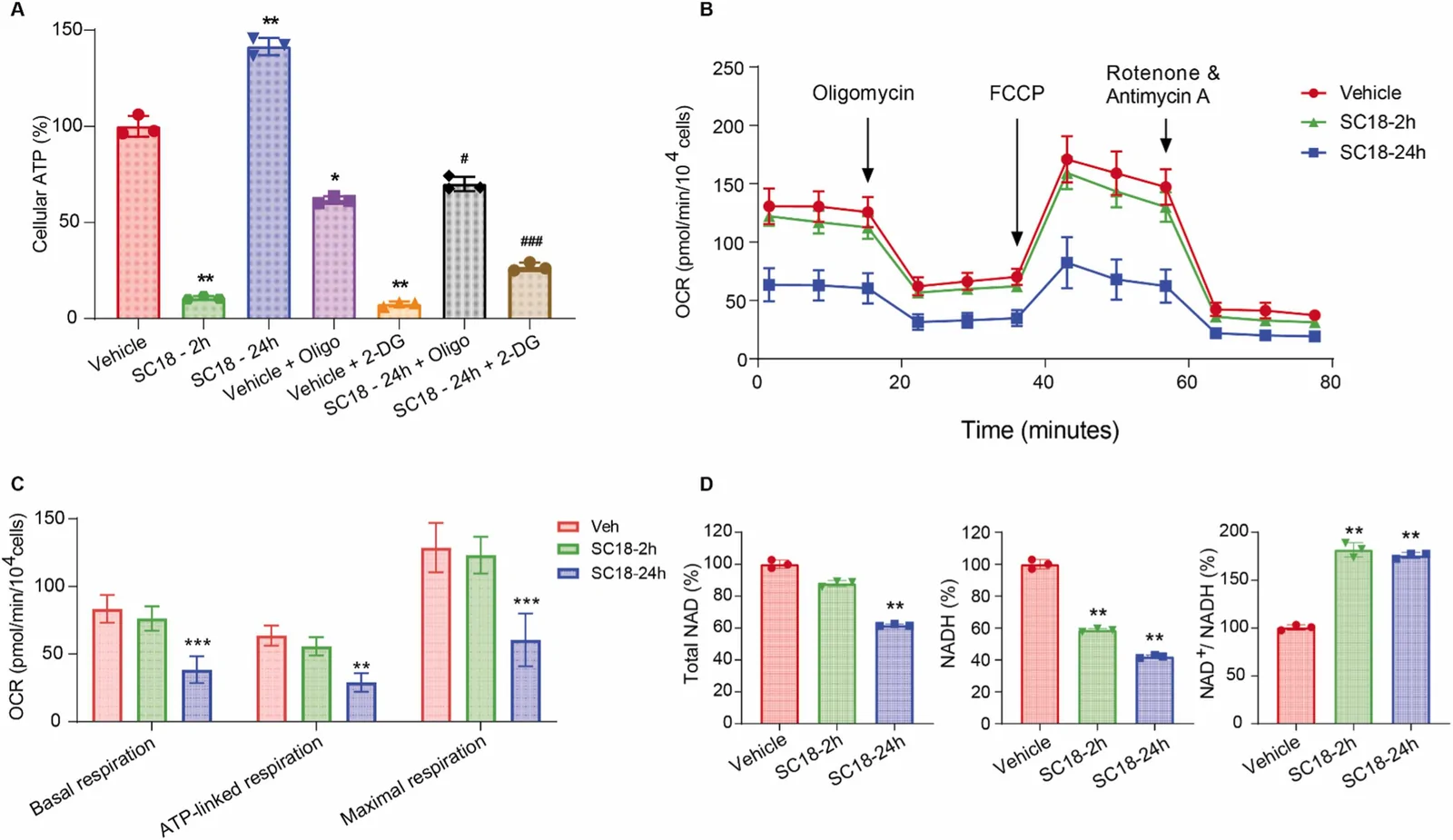
SC18 modulates cellular energy metabolism by altering ATP levels, mitochondrial respiration, and the NAD⁺/NADH ratio. (A) SC18 treatment (30 μM) reduced cellular ATP levels at 2 h, but led to a marked increase at 24 h, indicating a time-dependent shift in energy metabolism. Treatments with oligomycin (ATP synthase inhibitor) and 2-deoxyglucose (glycolysis inhibitor) show that both oxidative phosphorylation and glycolysis contribute to total ATP levels. (B–C) Oxygen consumption rate (OCR) analysis using Seahorse assay showed that SC18 had no major effect at 2 h, but decreased basal, ATP-linked, and maximal respiration after 24 h, indicating impaired mitochondrial function. (D) SC18 decreased total NAD^+^ and NADH levels at both 2 and 24 h, with a more pronounced reduction in NADH, resulting in an increased NAD⁺/NADH ratio-consistent with suppressed TCA cycle activity. Data represent mean ± S.D. of three independent experiments. Statistical significance was assessed by Student’s *t-test* or ANOVA. *: *p* < 0.05 and **: *p* < 0.01 compared to Vehicle. ^#^: *p* < 0.05 and ^###^: *p* < 0.001 compared to SC18-24 h. Abbreviations: ATP: adenosine triphosphate; NADH: nicotinamide adenine dinucleotide; NAD+: nicotinamide adenine dinucleotide; Oligo: oligomycin; 2-DG: 2-deoxy-D-glucose; FCCP: carbonyl cyanide 4-(trifluoromethoxy) phenylhydrazone.

**Fig. 5:**
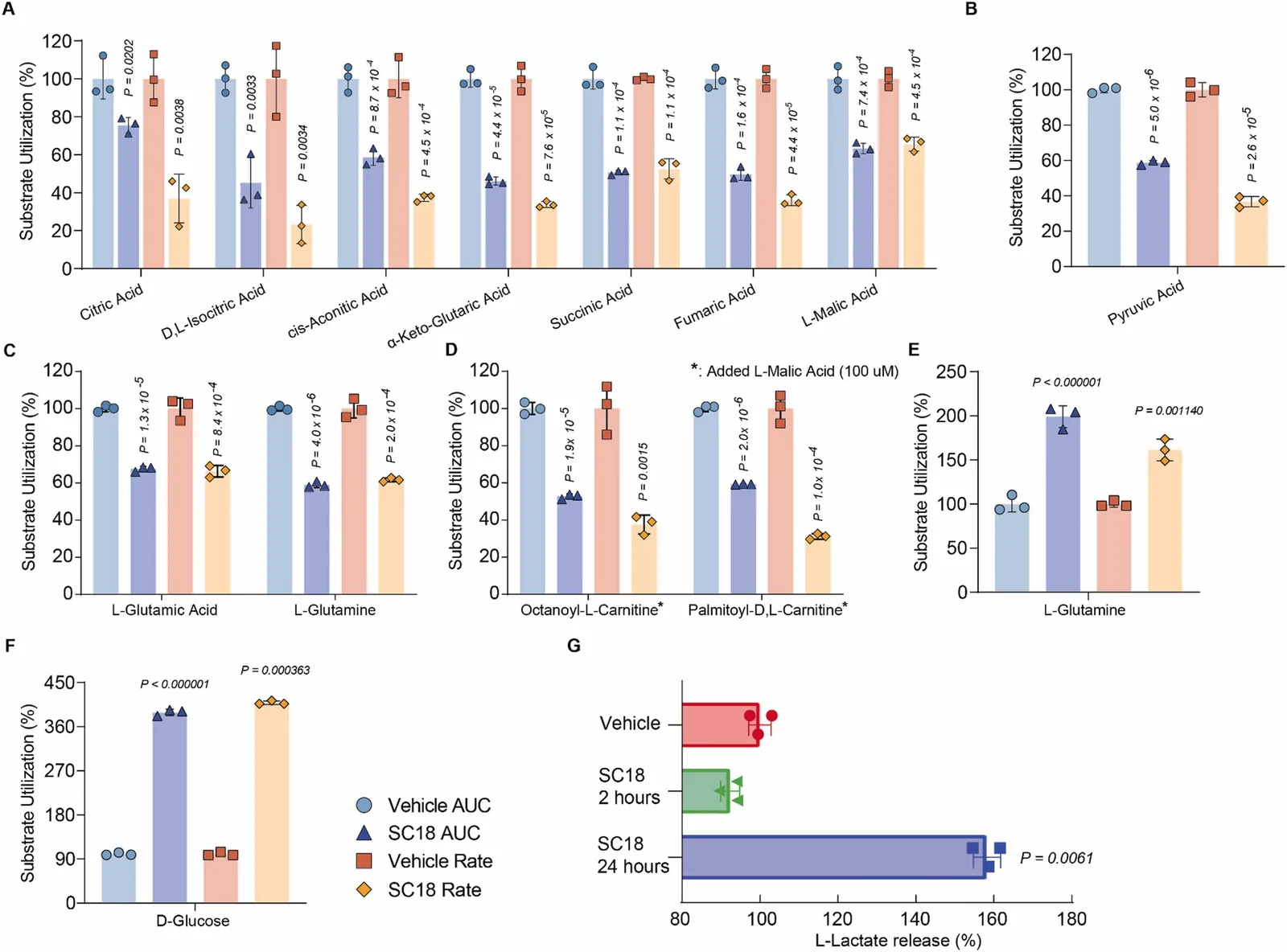
SC18 decreases mitochondrial substrate utilization and increases the cellular uptake of glucose and glutamine, enhancing glycolysis. (A–D) SC18 treatment (30 μM, 24 h) reduced mitochondrial substrate utilization in permeabilized Huh7 cells, as measured by Odin technology. SC18 inhibited the uptake and exchange of key tricarboxylic acid (TCA) cycle intermediates (A) pyruvate, (B) glutamate and glutamine, (C) acyl-carnitines including octanoyl-L-carnitine and palmitoyl-D,L-carnitine (D) even in the presence of L-malic acid. (E–F) In intact cells, SC18 induced a compensatory increase in the cellular uptake of glutamine (E), and glucose (F), indicating metabolic reprogramming. Substrate utilization was measured as area under the curve (AUC) and rate. (G) SC18 enhanced glycolytic flux, evidenced by increased L-lactate release after 24 h. Together, these findings indicate that SC18-induced VDAC1 inhibition disrupts mitochondrial metabolite flux, prompting a metabolic shift toward aerobic glycolysis to sustain ATP production. Data shown are mean ± S.D. of three independent experiments. Significance was assessed by Student’s *t-test*. Abbreviations: ATP: Adenosine triphosphate.

**Fig. 6:**
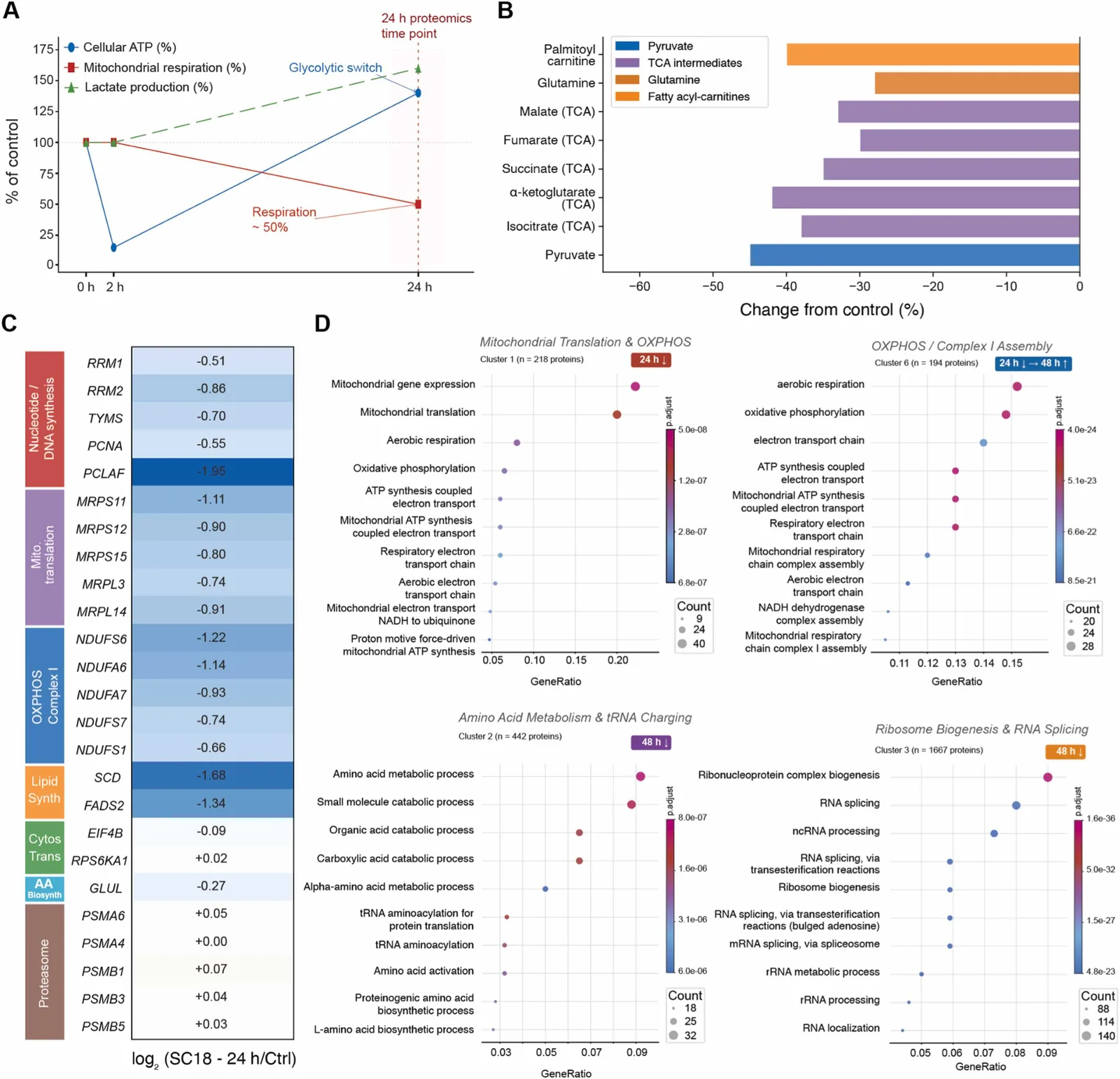
Correlation of metabolic changes induced by SC18 and proteome remodeling (A) Time-course measurements of cellular ATP levels, mitochondrial respiration, and lactate production in SC18-treated cells expressed as a percentage of untreated control. Within 2 h, cellular ATP drops sharply before recovering above baseline by 24 h, coinciding with a progressive increase in lactate production indicative of a glycolytic switch. Mitochondrial respiration declines steadily, reaching approximately 50% of control at 24 h. Both the 24- and 48-h time points were used for proteomics profiling (red dashed line). (B) Relative changes in intracellular metabolite levels at 24 h of SC18 treatment compared to control. Pyruvate is the most depleted metabolite (>60% reduction), followed by TCA cycle intermediates (isocitrate, α-ketoglutarate, succinate, fumarate, malate; 10–40% reduction), glutamine (∼35% reduction), and fatty acyl-carnitines including palmitoyl carnitine. The broad suppression of TCA intermediates is consistent with reduced mitochondrial substrate flux. (C) Heatmap of log₂ fold changes (SC18 24 h vs. control) for selected proteins grouped by functional category. Proteins involved in mitochondrial translation (MRPS/MRPL subunits), OXPHOS Complex I assembly (NDUFS/NDUFA subunits), nucleotide and DNA synthesis (RRM1/2, TYMS, PCNA, PCLAF), and lipid desaturation (SCD, FADS2) are the most downregulated. Proteasome subunits remain near baseline, suggesting selective rather than global proteome suppression. (D) Gene Ontology (GO) biological process enrichment from temporal proteomic cluster analysis. Cluster 1 (n = 218 proteins, downregulated at 24 h) is enriched for mitochondrial translation, OXPHOS, and ATP synthesis terms. Cluster 6 (n = 194 proteins, downregulated at 24 h and partially recovering at 48 h) is enriched for Complex I and respiratory chain assembly. Cluster 2 (n = 442 proteins, downregulated at 48 h) shows enrichment in amino acid metabolism and tRNA aminoacylation. Cluster 3 (n = 1667 proteins, downregulated at 48 h) is enriched for ribosome biogenesis and RNA splicing processes. Dot size reflects the number of proteins contributing to each term; color encodes adjusted p-value. Abbreviations: ATP: Adenosine triphosphate.

### SC18 Decreases ATP Production and Respiration, and Alters the NAD^+^/NADH Ratio

SC18 had a dual-time-course dependent effect on ATP production. 2 h treatment with SC18 decreased cellular ATP by ∼ 85% whereas 24 h treatment increased ATP by ∼ 40%. To determine the relative contribution of mitochondria and glycolysis to the overall production of ATP, we inhibited the ATP synthase with oligomycin (Oligo), and glycolysis with 2-deoxyglucose (2-DG). In separate 24 h experiments, both Oligo and 2-DG effectively decreased ATP indicating that maintenance of cellular ATP depends both on mitochondria and aerobic glycolysis (Fig. 4A, B). Moreover, we observed a dual-time-course effect of SC18 on respiration. 2 h treatment did not change basal, ATP-linked or maximal respiration as assessed by Seahorse assay. By contrast, 24 h treatment with SC18 decreased all basal, ATP-linked and maximal respiration by a minimum of 50%, indicating a direct effect of SC18 on oxidative phosphorylation (Fig. 4C). Additionally, SC18 at 2 h and 24 h decreased both Nicotinamide adenine dinucleotide (NAD^+^) and NADH. However, NADH decreased more than NAD^+^ causing the NAD^+^/NADH ratio to increase (Fig. 4D). These findings are consistent with diminished function of the TCA cycle.

### SC18 Reduces Mitochondrial Substrate Uptake and Induces Compensatory Glycolysis

To determine the effect of SC18 on the mitochondrial uptake and exchange of pyruvate, glutamine, and fatty acyl-CoAs, and the exchange of metabolic intermediaries of the TCA cycle between mitochondria and cytosol, we permeabilized the plasma membrane with saponin using Odin technology (Odin™, Biolog), which monitors cell substrate utilization rates. SC18 decreased the utilization of pyruvate and the TCA cycle intermediaries: D-L isocitric, cis-aconitic, α-keto-glutaric, succinic, fumaric and malic (Fig. 5A, B). In addition, SC18 decreased the utilization of glutamine, octanoyl-L-carnitine and palmitoyl-D-L-carnitine (Fig. 5C, D). These findings are consistent with a decreased exchange of metabolites between the cytosol and mitochondria caused by VDAC closure by SC18 for 2 h that inhibited oxidative phosphorylation and ATP synthesis. However, when treatment was sustained for 24 h, the increase in cellular ATP (Fig. 4A-B) is explained by the increased cellular uptake of glutamine and glucose by ∼300 % and 100 %, respectively (Fig. 5E, F), as we determined using Odin technology in intact cells. The augmented uptake of glucose indicates a compensatory effect caused by the switching to aerobic glycolysis. If mitochondria do not produce enough ATP as in this case because of VDAC1 closure, cells catabolize more glucose to lactate in the cytosol. This fact was clearly demonstrated by the ~ 60 % increase in lactate production (Fig. 5G).

## DISCUSSION

Previously, we identified SC18 through computational analysis as a ligand targeting the NADH-binding pocket of VDAC1. In the present study, we employed an integrative biophysical approach to define its binding mode and functional consequences.

NMR spectroscopy and molecular dynamics (MD) simulations demonstrated at atomic resolution that SC18 binds within the VDAC1 lumen at a site partially overlapping the NADH-binding pocket, consistent with steric interference within the pore. Notably, ligand engagement was enhanced in lipid bilayer nanodiscs compared to detergent micelles, indicating that a native-like membrane environment promotes productive binding and is consistent with the micromolar activity observed in cells (Heslop et al., 2022, Hiller et al., 2008). Comparison of AlphaFold3 co-folding and MD simulations provides complementary, model-dependent insight into potential ligand co-binding modes. In the AlphaFold3 prediction, SC18 and NADH occupy distinct regions within the VDAC1 lumen and are not positioned as closely adjacent as in the MD-derived model. By contrast, MD simulations sample configurations in which the two ligands are accommodated simultaneously within the pore in closer proximity, with NADH adopting a shifted position relative to the experimentally derived NMR model. Despite these differences in predicted geometry, both approaches support the possibility that SC18 and NADH can be present simultaneously within the channel lumen. Consistent with this, MD-based pore geometry analysis indicates that the dual-ligand condition is associated with a stronger local constriction than either ligand alone, suggesting additive steric effects. These observations should be interpreted cautiously given their computational nature, but they provide a structural framework consistent with the reduced conductance observed experimentally, rather than a predictive model of gating. Electrophysiological studies, however, provided direct functional evidence for the effect of SC18 on VDAC1 conductance. NADH alone partially decreased VDAC1 conductance, and subsequent addition of SC18 further suppressed the current, yielding a low-conductance state. In light of the structural models and pore-geometry analysis, this additional reduction is consistent with simultaneous occupancy of the VDAC1 lumen by NADH and SC18, leading to increased steric restriction within the pore. Although the precise co-binding arrangement remains model-dependent, the data support pore occlusion as a plausible basis for the reduced conductance, rather than a large-scale conformational gating transition.

Notably, proteomic analyses of SC18-treated cells showed upregulation of VDAC isoforms alongside enrichment of mitochondrial proteins, consistent with a compensatory response to impaired metabolite flux and reinforcing mitochondria as the primary site of SC18 action. This cellular response aligns with AlphaFold3 co-folding predictions, which indicate that SC18 engages a structurally conserved binding pocket across VDAC1, VDAC2, and VDAC3. The preservation of this intra-barrel binding site among isoforms supports the notion that SC18 can broadly target VDAC channels, providing a structural basis for the observed upregulation of multiple VDAC isoforms in response to treatment.

With the channel-level mechanism established, we next examined the impact of VDAC1 inhibition by SC18 on cancer cell metabolism. Prior work demonstrated that SC18 causes mitochondrial dysfunction and induce cell death in human hepatocarcinoma cells, both as a standalone treatment and synergistically with FDA-approved drugs for hepatocellular carcinoma such as sorafenib (Heslop et al., 2022, Ventura et al., 2023). To bridge the mechanistic gap between channel closure and cell death, we combined readouts of mitochondrial and cellular metabolism such as ATP, NADH, respiration and lactate release, with the novel Odin technology. Odin provides functional metabolomics data allowing the identification of both mitochondrial and cellular utilization of substrates. Short treatment for 2 h with SC18 decreased cellular ATP and respiration indicating an immediate impairment of oxidative phosphorylation. The decrease in oxidative phosphorylation was associated with a markedly smaller mitochondrial uptake of key fuels for the TCA cycle such as pyruvate and glutamine and the consequent decreased levels of several TCA cycle intermediates. Thus, the overall inhibition of mitochondrial metabolism was caused by the closure of VDAC1 that impeded the normal flux of oxidizable metabolites to support oxidative phosphorylation. This suggests that the mechanism triggering cell death after VDAC closure could be the diminished mitochondrial generation of metabolic intermediaries for the biosynthesis of macromolecules and not the failure in producing ATP (Ventura et al., 2023). Consistent with this hypothesis, we found that several enzymes involved in fatty acid metabolism, nucleotide and DNA synthesis, and protein translation were downregulated. Concomitant with the mitochondrial suppression occurring 24 h after treatment, we found a compensatory increase in the uptake of glutamine and glucose at the cellular level. Increased uptake of glucose in intact cells treated with SC18-treated cells in parallel with decreased oxidation of pyruvate in mitochondria, strongly suggest that cytosolic aerobic glycolysis was augmented to compensate for the inhibition of oxidative phosphorylation. This fact was also confirmed by the increased lactate production typical of the Warburg phenotype. Of note, lactate does not accumulate inside the cells and is released to the medium, where we measured this metabolite. In cancer cells, both mitochondrial metabolism and enhanced aerobic glycolysis coexist in a dynamic equilibrium (Maldonado, 2017). One critical factor influencing the relative contribution of each metabolism to the overall ATP production is the availability of substrates to be oxidized. Thus, the shift observed 24 h after VDAC1 inhibition, is a typical example of survival adaptability of cancer cells to the energetic restriction imposed by the treatment.

In essence, our data collectively indicate that SC18 forces a metabolic trade-off with cells maintaining ATP production through aerobic glycolysis, while slowly starving from lack of biosynthetic intermediaries. The biosynthetic failure explains that cell death occurs days after treatment with SC18 as shown before (Ventura et al., 2023), because it is not caused by the lack of ATP caused by mitochondrial dysfunction after VDAC closing, but because of the impaired ability of surviving cells to generate biomass for daughter cells.

Together, our findings establish a mechanistic framework for targeting VDAC as a novel anticancer strategy, one that disrupts mitochondrial substrate flux, triggers a compensatory shift to glycolysis, and ultimately impairs the biosynthetic capacity essential for tumor cell survival and proliferation. This study provides proof-of-concept for pharmacologically targeting VDAC in cancer cells and suggests that combining VDAC inhibitors with chemotherapeutic agents might overwhelm the metabolic plasticity of tumors. Moving forward, further studies should examine if this metabolic rerouting after VDAC inhibition occurs in different cancer types. Ultimately, exploiting this dual metabolic vulnerability could prove to be a powerful new strategy in the fight against cancer.

## MATERIALS AND METHODS

### Expression and Purification of Voltage Dependent Anion Channel 1

All VDAC isoforms were expressed using the same protocol. hVDAC-1 WT (VDAC-1 with C-terminal HisTag) and hVDAC-1 E73V (VDAC-1 E73V with C-terminal HisTag) as well as hVDAC-1 Avi (VDAC-1 with C-terminal AviTag) were expressed from pET21a-based plasmids. The plasmid was transformed to *E. coli* Lemo BL-21(DE3) cells using a heat-shock protocol^8^. After a 5 mL overnight preculture, cells were grown in LB or isotope-labeled medium to an OD_600_ of 0.6 and then induced with 1 mM IPTG. For [U-^2^H,^15^N]-labeling, a D_2_O-based M9 minimal medium supplemented with ^15^NH^4^Cl was used. After overnight expression at 37°C, cells were harvested by centrifugation and flash frozen until further use. Thawed cell pellets were resuspended and incubated in 30 mL of resuspension buffer (10 mM Tris-HCl, 1 mM EDTA, pH 7.0) per liter of expression. Protease inhibitor cocktail (ROCHE cOmplete Mini, EDTA-free) DNAse (Applichem) and lysozyme were supplemented to the sample and incubated for 30 min at 4°C. Cells were lysed by sonication and centrifuged at 30,000 xg for 30 min. The pellet was resuspended with a detergent containing buffer (10 mM Tris- HCl, 1 mM EDTA, 2% Triton X-100, pH 7.0) incubated at room temperature for 1 h and centrifuged again at 30,000 g for 30 min. The pellet was washed with 3 mL of resuspension buffer, and was resolubilized into a binding buffer (50 mM Tris-HCl, 100 mM NaCl, 8 M Urea, 20 mM imidazole, pH 7.5). Cell debris were separated from the solubilized inclusion bodies by centrifuging the sample at 186,000 xg for 90 min. The supernatant was filtered with 0.8 µM pore size filters and taken for IMAC. A HisTrap column was connected to a peristaltic pump and IMAC was done by applying the sample to the column followed with an extensive wash with binding buffer (50 mM Tris-HCl, 100 mM NaCl, 8 M Urea, 20 mM imidazole, pH 7.5), and elution of the VDAC containing fractions with elution buffer (50 mM Tris-HCl, 100 mM NaCl, 8 M Urea, 250 mM imidazole, pH 7.5). Elution fractions were merged and dialyzed overnight against water at 4 °C. White protein precipitates were collected by centrifugation at 26,000 xg for 30 min and dissolved in GuHCl buffer (100 mM Napi, 100 mM NaCl, 6 M GuHCl, 5 mM DTT, 1 mM EDTA, pH 7.0). The unfolded protein was introduced to the pre-cooled refolding buffer (25 mM Napi, 100 mM NaCl, 1% LDAO, 5 mM DTT, 1 mM EDTA, pH 7.0) in a drop-wise manner while the sample was being stirred. After overnight stirring the sample was taken to dialysis in dialysis buffer (25 mM Napi, 1 mM EDTA, 5 mM DTT, pH 6.5). Proper refolding efficiency was obtained for dialyzing 80 mL of the protein sample/refolding buffer mixture against 5 L of dialysis buffer (25 mM Napi, 5 mM DTT, 1 mM EDTA, pH 6.5) overnight and for another 2 h the next day in a 5 L of dialysis buffer. Refolded protein was purified by ion exchange chromatography on a Hi-Trap SP HP (5 mL) column, using buffer A (25 mM Napi, 0.1% LDAO, 5 mM DTT, pH 6.5) and buffer B (25 mM Napi, 1 M NaCl, 0.1% LDAO, 5 mM DTT, pH 6.5). The protein was eluted with a gradient of buffer B. The pooled elution fractions were concentrated and used for reconstitution. Purified protein was flash frozen and stored at −80 °C.

### Expression and Purification of Membrane Scaffold Proteins

Expression and purification of all membrane scaffold proteins was performed as described (Bibow et al., 2020, Modaresi et al., 2024). The pET28a plasmid encoding MSP with N-terminal HisTag and cleavage site was transformed into *E. coli* BL21(DE3) cells. An overnight culture was inoculated into 1 L of LB in baffle flasks. Cells were grown at 37 °C until O.D_600_ of 0.7–0.8 and then induced with 0.5 mM of IPTG. 1 h after induction, the temperature was lowered to 28 °C and expression was continued for another 4 h. Cells were pelleted by centrifugation at 4000 xg for 20 min at 4 °C. Next, cells were resuspended in resuspension buffer (20 mM Tris-HCl, 500 mM NaCl, pH 8.0) supplemented with 5 mg of DNAse (Applichem) per liter of expression and 1% of Triton X-100, volumetrically. The mixture was incubated and shaken at 4 °C for 1 h. Cells were then sonicated with a Bronson sonifier for 20 min on 20% power of 2 s “on” followed by 2 s “off” pulses. The sonicated sample was centrifuged at 30,000 xg for 30 min at 4 °C after which the supernatant was filtered by syringe and non-pyrogenic 0.2 µM sterile filters. The filtered sample was then injected to an AKTA machine for Ni-NTA based purification. Two HisTrap columns (Cytiva - 2×5 mL) were connected and equilibrated with priming buffer (20 mM Tris-HCl, 500 mM NaCl, pH 8.0, 1% TritonX-100). The sample was applied to the column with a flowrate of 1 mL/min. The columns were then washed again with 100 mL of priming buffer. Next, 100 mL of cholate buffer (20 mM Tris-HCl, 500 mM NaCl, pH 8.0, 50 mM sodium cholate) was introduced to the columns. Afterwards, another 100 mL of resuspension buffer was applied to the column to prepare the column for gradient elution. Unspecific bound impurities were washed out from the column by wash buffer (20 mM Tris-HCl, 500 mM NaCl, pH 8.0, 50 mM Imidazole) and the His tagged MSP was eluted by gradient application of elution buffer (20 mM Tris-HCl, 500 mM NaCl, pH 8.0, 500 mM Imidazole). The eluted fractions were quickly exchanged to MSP final buffer (20 mM Tris-HCl, 100 mM NaCl, pH 7.4). The HisTag was cleaved by introduction of TEV protease in 1:100 ratio while dialyzing at 4 °C. The sample was then subjected to a reverse HisTrap to remove the TEV protease and the cleaved HisTag. A final Size Exclusion Chromatography (SEC) was done in SEC buffer (20 mM Napi, 100 mM NaCl, pH 7.0) as the final step. The protein was then aliquoted into 500 μL with a concentration of 200 μM and stored at −80 °C until further use.

### Reconstitution of Voltage Dependent Anion Channel 1 in MSP1D1 Nanodiscs

VDAC reconstitution was performed based on previously established protocols (Bibow et al., 2020). The reconstitution of VDAC into MSP1D1 was mediated either by 1,2-dimyristoyl-sn-glycero-3-phosphocholine (DMPC 14:0) or mixed with Hexadecanoyl-2-(9Z-Octadecenoyl)-sn-Glycero-3-Phosphoglycerol (POPG 16:0 18:1) lipids. A fixed molar ratio of VDAC: SP: DMPC: sodium cholate (1: 4: 200: 400) was used resulting in 25 μM: 100 μM: 5 mM: 10 mM, respectively. Samples were mixed in nanodisc buffer (25 mM Napi, 100 mM NaCl, 5 mM DTT, pH 7.0) with Bio-beadsTM SM-2 Adsorbent Media (Bio-RAD) for cholate and LDAO removal, mediating nanodisc reconstitution process at room temperature on an orbital shaker overnight forming an assembled nanodisc complex. Supernatant was run on a Size Exclusion Chromatography column (superdex 16600 s200pg 25 mL).

### Mass Photometry

Mass photometry was performed to determine the molecular mass of VDAC1 reconstituted in nanodiscs. Measurements were conducted on a Refeyn OneMP instrument using VDAC1-nanodisc complexes at a final concentration of 10 nM in SEC buffer (25 mM Napi, 100 mM NaCl, 5 mM DTT, pH 7.0). All measurements were performed immediately after purification and nanodisc assembly. Coverslips were cleaned by sequential rinsing with Milli-Q water and isopropanol, followed by drying under a nitrogen stream. For each measurement, 18 µL of SEC buffer was applied to the coverslip to establish focus, followed by 2 µL of protein sample. Data were acquired over 60 s using Refeyn AcquireMP software under standard acquisition settings. Recorded videos were analyzed with Refeyn DiscoverMP software. Molecular masses were calculated by converting interferometric contrast values using a standard calibration curve generated with protein standards under identical buffer conditions.

### Cryo-Electron Microscopy

An aliquot of VDAC1 WT reconstituted in MSP1D1 (DMPC14:0) nanodiscs in 25 mM Napi, 5 mM DTT, pH 7.0, 100 mM NaCl at a concentration of 2 mg/mL (1 Abs = 1 mg/mL) was applied to a Quantifoil R2/1 holey carbon copper grid (Quantifoil Micro Tools) vitrified by plunging into liquid ethane using a Lacie vitrification robot. A dataset was collected using a Glacios electron microscope (ThermoFisher) operated at 200 kV, equipped with a Quantum K3 direct electron detector (DED, Gatan) operating in counting mode using SerialEM. Images were acquired at 0.8–2.5 μm defocus and a nominal magnification of 45,000×, corresponding to a pixel size of 0.878 Å. 6350 movies were collected with a total dose of approximately 53.32 e/Å^2^ per 3.995 s exposure, fractionated over 40 frames.

### Electron Microscopy Data Processing

Micrographs were corrected for beam-induced drift using patch motion correction, and contrast transfer function (CTF) parameters were estimated for each micrograph using Patch CTF in cryoSPARC. Particles were picked using the blob picker function in cryoSPARC and subjected to iterative reference-free 2D classification. Following exposure curation, 373,906 particles were selected and used for ab initio 3D reconstruction. Subsequent heterogeneous refinement resolved a few conformational classes, and the best-populated class was carried forward for further refinement. Non-uniform (NU) refinement was applied iteratively, interspersed with additional rounds of ab initio reconstruction and heterogeneous refinement to improve map quality. Angular distribution analysis confirmed adequate sampling of particle orientations. A representative summary of the data processing workflow is provided in Supplementary Figure 5. Initial model fitting was performed by manually docking the VDAC1 NMR structure (PDB: 6TIQ) into the EM density map in UCSF ChimeraX. All structural models were visualized using ChimeraX.

### Solution NMR-Transverse Relaxation Optimized Spectroscopy Experiments and Data Analysis

VDAC-1 protein for NMR spectroscopy were expressed in M9 medium, supplemented with perdeuterated water and 1.0 g/L ^15^N-ammonium chloride. Proteo-nanodiscs were purified in 25 mM Napi, pH 7.0, 5 mM DTT, 100 mM NaCl were concentrated to a final concentration of 18 mg/mL (1 Abs = 1 mg/mL). 2D [^15^N, ^1^H]-TROSY-HSQC experiments were measured on a 600 MHz Bruker spectrometer equipped with a cryogenic probe. 170 transients were accumulated at temperature of 37 °C for the initial apo sample, where the number of scans were increased accordingly upon titration step to re-cover the protein dilution effect with 1024 and 256 complex points in the ^1^H and ^15^N dimensions, respectively. Assignment of hVDAC-1 WT in nanodiscs (BMRB: 28053) was performed as described in the former section and was used for structural analysis. SC18 was prepared as a 60 mM stock solution in DMSO-d6 and introduced into ∼300 µL VDAC1 samples reconstituted in LDAO micelles for 2D ^1^H–^15^N TROSY-HSQC experiments. A matched volume of DMSO-d6 added to the same protein-containing buffer without SC18 was used as the vehicle control. Chemical shift perturbations (CSPs) were calculated using the weighted formula: CSP = √[(Δδ^1^H)^2^ + (0.154 × Δδ^15^N)^2^], where Δδ^1^H and Δδ^15^N (DMSO-d6 only) are the differences in ^1^H and ^15^N chemical shifts (in ppm) between SC18-bound and vehicle conditions, respectively; the weight factor 0.154 reflects the gyromagnetic ratio correction for ^15^N at 600 MHz. Residues with CSP values exceeding mean + 2 standard deviations of the dataset were defined as significantly perturbed. Peak intensities were extracted from 2D ¹H–¹⁵N TROSY-HSQC spectra as peak heights using CCPnmr. The vehicle (DMSO-d6) and SC18-bound (6 mM SC18) spectra were processed with differing Bruker NC_proc scaling parameters; raw CCPN heights were therefore multiplied by a correction factor of 2^(−3)/2^(−1) = 0.25 before computing ratios. The signal attenuation ratio I_bound_/I_free_ was defined as the corrected SC18-bound peak height divided by the corrected vehicle peak height. Signal attenuation was quantified as the peak intensity ratio I_bound_/I_free_, calculated from the heights of resolved backbone amide resonances in identically processed SC18-bound and vehicle spectra. Residues with I_bound_/I_free_ < 0.1 were classified as undergoing significant signal broadening, indicative of direct involvement in the SC18 binding interface.

### Solution NMR-Saturation Transfer Difference Experiments

Experiments were conducted using a Bruker Avance III 600 MHz spectrometer. VDAC1 was reconstituted either in LDAO micelles or in MSP1D1 nanodiscs containing DMPC (14:0) lipids. Final sample conditions consisted of 5 µM VDAC1 and 500 µM SC18 in 20 mM phosphate buffer (pH 7.4), 150 mM NaCl, and 10% D₂O for lock. Selective saturation of protein resonances was achieved using a train of Gaussian-shaped pulses (50 ms duration, total saturation time = 2 s) applied at 0.5 ppm (on-resonance) and 40 ppm (off-resonance). Water suppression was implemented using excitation sculpting. A total of 512 scans were recorded for each spectrum with a relaxation delay of 4 s. Spectral subtraction was performed to generate the STD difference spectra. For quantitative comparison across conditions, all spectra (off-resonance, on-resonance, and STD) were noise-normalized by dividing by the root-mean-square noise of the corresponding off-resonance spectrum measured in the noise-only region (8.5–9.0 ppm), yielding a common noise standard deviation of σ = 1 across all panels. Signal-to-noise ratios (SNR) for the STD difference spectra were calculated as peak amplitude divided by the noise standard deviation of the corresponding STD spectrum. Integration of STD signals was performed over three defined windows: aromatic/olefinic (8.85–6.57 ppm), olefinic/OCH₂ (6.50–4.58 ppm), and aliphatic (4.50–0.16 ppm), using the trapezoidal rule. An MSP1D1 nanodisc sample lacking VDAC1 (−VDAC control) was included as a specificity control. A ∓3σ noise band is displayed on all STD panels to guide interpretation of signal significance. The temperature was 37 °C for all STD experiments.

### Solution NMR-TRACT Experiments

[¹⁵N,¹H]-TROSY for rotational correlation times experiments were performed at 37 °C on a Bruker Avance III HD 600 MHz spectrometer equipped with a cryogenically cooled probe. The TROSY for rotational correlation times pulse sequence was used to selectively measure the slow-relaxing α-spin (R₂⁺) and fast-relaxing β-spin (R₂⁻) states of ¹⁵N nuclei. Data were acquired with an increment of 600 μs, and the number of scans was adjusted to ensure sufficient signal-to-noise. Bulk relaxation rates were obtained by integrating the amide proton region (9.5–8.5 ppm) and fitting the decay curves to a single exponential model. The difference ΔR₂ = R₂⁻ - R₂⁺ was used to calculate the rotational correlation time (τ_c_) according to established protocols.

### Thermostability Analysis of Protein Samples

Thermal denaturation experiments were performed using a Nanotemper Prometheus NT.48 instrument. Samples were held in standard capillaries and subject to a continuous thermal ramp from 20°C-95°C at a rate of either 0.5 or 1°C /min. Denaturation was monitored by intrinsic protein fluorescence using excitation at 280 nm and measuring emission at 330 and 350 nm. Denaturation curves were obtained by plotting the ratio of emission intensity at 350 nm to emission intensity at 330 nm as a function of temperature. The midpoint of thermal denaturation was obtained from the peak in the first-derivative of the curve using the automated algorithm in the instrument software.

### Molecular Dynamics Simulations

To investigate the impact of SC18 and NADH complexation on the structural characteristics of VDAC, full-atomistic molecular dynamics (MD) simulations were performed. The NMR structural model of VDAC (PDB code: 6TIQ) was obtained from the Protein Data Bank. The standard protonation states of all VDAC amino acids at pH 7.0 were determined and verified using the H^++^ web server (Anandakrishnan et al., 2012). Complexes of VDAC with SC18 and NADH were generated using AutoDock Vina software (Trott and Olson, 2010). The docking results with the highest binding affinities were selected for subsequent MD simulations. The receptor, in both ligand-bound and ligand-free forms, was embedded in a Dimyristoylphosphatidylcholine (DMPC) lipid bilayer (∼88 × 82 × 105 Å³). The system was then solvated with TIP3P water, and periodic boundary conditions were applied in all directions (Jorgensen et al., 1983). Force field parameters were assigned using the General Amber Force Field (GAFF) for SC18, LIPID17 for lipid bilayer and the FF14SB force field for the receptor and NADH (Maier et al., 2015, Wang et al., 2004, Skjevik and Madej, 2016). Atomic charges for SC18 were derived using the CHelpG method at the B3LYP/6–31+G(d) level of theory with Gaussian 16 (Frisch et al., 2016, Zhao and Truhlar, 2006). All MD simulations were performed using the Amber 22 software package (Case et al., 2020).

The simulations began with energy minimization over 100,000 steps. The systems were then heated from 0 to 300 K under an NVT ensemble over 10 ns (1 fs time step) with a force constant of 2.5 kcal·mol⁻¹·Å⁻² applied to solute atoms. This was followed by a 20 ns equilibration phase under NPT conditions (300 K, 1 bar, 1 fs time step) without positional restraints. Finally, 200 ns production runs (2 fs time step) were conducted under an NPT ensemble. As a control, simulations of ligand-free VDAC were also performed to assess the effects of ligand binding on VDAC’s structural stability and dynamic behavior. During equilibration and production, temperature and pressure were regulated using the Langevin thermostat (1 ps⁻¹ collision frequency) and the Berendsen barostat (1 ps relaxation time), respectively (Sindhikara et al., 2009, Uberuaga et al., 2004). Long-range electrostatic interactions were calculated using the particle mesh Ewald (PME) method with a 12 Å cutoff (Darden et al., 1993). The SHAKE algorithm was applied to constrain bonds involving hydrogen atoms (Ryckaert et al., 1977). Binding energies for the complexation process were computed using the molecular mechanics generalized Born surface area (MM-GBSA) approach (Miller et al., 2012). The final 50 ns of the production phase were used to evaluate binding energies. All simulations were performed in three independent replicates for 200 ns.

### Pore Geometry Analysis

The pore geometry of the receptor was analyzed using the MDTraj package.^1^ The MD trajectories were used to evaluate the pore radius along the channel axis in both the absence and presence of the ligands. For each system, the radius profile was calculated along the pore coordinate to identify constriction regions and assess changes in channel geometry induced by ligand binding. The resulting profiles were averaged over the sampled configurations to obtain a representative description of the pore architecture for each condition.

### AlphaFold Structural Predictions of Voltage Dependent Anion Channel–Holo Complexes

Human VDAC isoforms were modeled using the UniProt reference sequences for VDAC1 (P21796), VDAC2 (P45880), and VDAC3 (Q9Y277). To evaluate potential holo-complex formation, structure predictions were generated for each VDAC isoform in the presence of small-molecule ligands using AlphaFold 3. The ligand corresponding to SC18 was provided as a SMILES string (SC18:O=c1[nH]c2ccc([N+](=O)[O-])cc2c(=O)n1-c1cc(C(F)(F)F)ccc1Cl and NADH: O=C(N)C1CC=C[N](C=1)[C00H]2O[C00H]([C00H](O)[C0H]2O)COP([O-])(=O)OP(=O)([O-])OC[C0H]5O[C00H](n4cnc3c(ncnc34)N)[C0H](O)[C00H]5O) and included as a non-protein component in the modeling input. Additional runs were performed with either a second ligand alone or with both ligands present simultaneously, generating VDAC1, VDAC2, and VDAC3 complex models for the ligand 1 (SC18), ligand 2(NADH), and ligand 1 + ligand 2 conditions. For each modeling job, AlphaFold 3 was run with stochastic sampling, and the top-ranked model was selected on the basis of the internal ranking score. The best-scoring models retained for analysis were: VDAC1 + ligand 1 (ranking score 0.9337), VDAC2 + ligand 1 (0.9489), VDAC3 + ligand 1 (0.9291), VDAC1 + ligand 2 (0.7407), VDAC2 + ligand 2 (0.7341), VDAC3 + ligand 2 (0.8076), VDAC1 + ligand 1 + ligand 2 (0.8036), VDAC2 + ligand 1 + ligand 2 (0.8606), and VDAC3 + ligand 1 + ligand 2 (0.8789). Across jobs, the highest-ranking seed/sample combination was retained as the representative complex for downstream structural inspection. Predicted structures were exported as coordinate files and visually inspected to assess ligand placement, overall compatibility of the ligand-bound state with the VDAC barrel architecture, and differences among VDAC isoforms. Comparative analysis focused on the relative positioning of SC18 within the predicted binding environment and on whether co-modeling with the second ligand altered the predicted ligand pose or local protein conformation.

### Electrophysiology

A bilayer was formed using 1,2-diphytanoyl-sn-glycerol-3-phosphocholine (DiPhPC, Avanti Polar Lipids), at the 50–100 µm diameters apertures of Teflon partition as previously described (Amodeo et al., 2021). Briefly, the aperture was pre-treated with 25 mg/mL of DiPhPC in decane and allowed to dry. Bilayers were formed using the painting method with the same lipid solution. Cuvettes were filled with the recording solution (150 mM KCl, 20 mM HEPES, pH 7.4) on both sides of the partition. Ion currents were measured using standard Ag-AgCl electrodes (World Precision Instruments) that were placed in each side of the cuvette without use of agar bridges. Single channels activity were induced by adding the protein to the cis side of the chamber (the side connected to the ground electrode). Spontaneous channel insertion was typically obtained under an applied voltage of 20 mV. Conductance measurements were performed using an eONE amplifier (Elements) with a sampling rate of 10 kHz (809.1 µs interval).

### Cell Culture

Human Huh7 cells were grown in RPMI medium supplemented with 10% FBS (Fisher Scientific; Cat#11560636), 2 mM L-glutamine (Fisher Scientific; Cat#BE17–605E) and 100 units per mL of penicillin/streptomycin (Thermo Fisher; Cat#15140122) and incubated at 37 °C in 5% CO_2_. In IC_50_ determination, cells were seeded at a density of 4000 per well in 96-well plates and after a day of growth, treated with the molecules for 48 h. Thereafter cell viability was measured using CellTiter-Blue Cell Viability Assay (Promega; Cat#G8081) according to the manufacturer protocol.

### Proteomics Sample Preparation

Sample preparation was performed according to our recent protocol (Saei et al., 2019). In brief, cells were seeded at a density of 250,000 per well and allowed to grow for 24 h in 4 biological replicates. Next, cells were either treated with vehicle or SC18 at 30 µM. After treatment, cells were collected, washed twice with PBS and then lysed using 8 M urea (Sigma; Cat#U5378), 1% SDS, and 50 mM Tris at pH 8.5 with protease inhibitors (Sigma; Cat#05892791001). The cell lysates were subjected to 1 min sonication on ice using Branson probe sonicator and 3 s on/off pulses with a 30% amplitude. Protein concentration was then measured for each sample using a BCA Protein Assay Kit (Thermo; Cat#23227). 50 µg of each sample was reduced with DTT (final concentration 10 mM) (Sigma; Cat#D0632) for 1 h at room temperature. Afterwards, iodoacetamide (IAA) (Sigma; Cat#I6125) was added to a final concentration of 50 mM. The samples were incubated at room temperature for 1 h in the dark, and the reaction was quenched by addition of 10 mM DTT. After precipitation of proteins using methanol/chloroform, the semi-dry protein pellet was dissolved in 25 µL of 8 M urea in 20 mM EPPS (pH 8.5) (Sigma; Cat#E9502) and was then diluted with EPPS buffer to reduce urea concentration to 4 M. Lysyl endopeptidase (LysC) (Wako; Cat#125–05061) was added at a 1: 75 w/w ratio to protein and incubated at room temperature overnight. After diluting urea to 1 M, trypsin (Promega; Cat#V5111) was added at the ratio of 1: 75 w/w and the samples were incubated for 6 h at room temperature. Acetonitrile (Fisher Scientific; Cat#1079–9704) was added to a final concentration of 20% v/v. TMTpro 16plex reagents (Thermo) were added 4x by weight to each sample, followed by incubation for 2 h at room temperature. The reaction was quenched by addition of 0.5% hydroxylamine (Thermo Fisher; Cat#90115). Samples were combined, acidified by trifluoroacetic acid (TFA; Sigma; Cat#302031-M), cleaned using Sep-Pak (Waters; Cat#WAT054960) and dried using a DNA 120 SpeedVac™ concentrator (Thermo). Samples were then resuspended in 20 mM ammonium hydroxide and separated into 96 fractions on an XBrigde BEH C18 2.1 × 150 mm column (Waters; Cat#186003023), using a Dionex Ultimate 3000 2DLC system (Thermo Scientific) over a 48 min gradient of 1–63%B (B = 20 mM ammonium hydroxide in acetonitrile) in three steps (1–23.5%B in 42 min, 23.5–54%B in 4 min and then 54–63%B in 2 min) at 200 µL min^−1^ flow. Fractions were then concatenated into 12 samples in sequential order (e.g. 1, 13, 25, and so on). After drying and resuspension in 0.1% formic acid (FA), each fraction was analyzed.

### LC-MS Analysis

Samples were loaded with buffer A (0.1% FA in water) onto a 50 cm EASY-Spray column (75 µm internal diameter, packed with PepMap C18, 2 µm beads, 100 Å pore size; Cat#ES803) connected to a nanoflow Dionex UltiMate 3000 UPLC system (Thermo) and eluted with a buffer B (98% ACN, 0.1% FA, 2% H2O) gradient from 2% to 35% of at a flow rate of 250 nL min^−1^. Mass spectra were acquired with an Orbitrap Exploris 480 mass spectrometer (Thermo; Cat# IQLAAEGAAPFALGMBDK) in the data-dependent mode with MS1 scan at 70,000 resolution, and MS2 at 35,000, in the *m/z* range from 375 to 1400. Peptide fragmentation was performed via higher-energy collision dissociation (HCD) with energy set at 35 NCE.

### Protein Identification and Quantification

Thermo Xcalibur 4.0 was used to control and process the liquid chromatography-mass spectrometry data. The raw liquid chromatography-mass spectrometry data were analyzed by Proteome Discoverer version 2.5.0.400 with TMTpro 16plex as an isobaric labeling mass tag. The Sequest search engine matched MS/MS data against the UniProt complete proteome database. Cysteine carbamidomethylation was set as a fixed modification, while oxidation (M) was selected as variable modifications. Trypsin was selected as enzyme specificity. No more than two missed cleavages were allowed. A 1% FDR was used as a filter at both protein and peptide levels. For all other parameters, the default settings were used.

### Network Mapping

GO pathway enrichment analysis for the top proteins was performed using StringDB v. 12. (Szklarczyk et al., 2025).

### Metabolic Profiling Using Phenotype Microarray Plates

Huh7 cells were treated with either vehicle or SC18 (30 μM) for 24 h using Biolog Phenotype Microarray (PM) PM-M1 & PM-M2 and MitoPlate S-1 assays (Biolog, Hayward, CA) to assess metabolic changes in intact and permeabilized cells, respectively. PM-M1 & PM-M2 plates are 96-well plates precoated with carbon- and nitrogen sources in the low millimolar range. Both assays use a tetrazolium-based redox dye as a final electron acceptor of the electron transport chain. Color changes of the dye allow characterize the kinetics of the utilization of the substrates. For PM-M1 & PM-M2 plates, cells were resuspended in IF-M1 medium supplemented with 5 % FBS, penicillin/streptomycin and 0.3 mM glutamine followed by inoculation of 20,000 cells per well. After incubation for 24 h at 37 °C in a 5 % CO_2_ incubator, Redox Dye MB was added to each well and the kinetic reading was immediately carried out in an Odin instrument (Biolog Inc., Hayward, CA) which uses a CCD camera to capture the purple color formation every 5 min at 37 °C, for 24 h. Background was corrected for the blank sample and the plateau values were calculated. To phenotype mitochondrial function, we used the MitoPlate S-1 (Biolog, Hayward, CA) which contains substrates exclusive to the tricarboxylic acid (TCA) cycle, amino acids, and a ketone body in three technical replicates. Cytosolic substrates (D-glucose, glycogen, D-glucose 1-phosphate, D-glucose 6-phosphate, 6-phospho-D-gluconate, and lactate) served as negative controls in the MitoPlate S-1. Pyruvate and some mitochondrial substrates (L-leucine, γ-aminobutyric acid, α-ketoisocaproic acid, acetylcarnitine, octanoylcarnitine and palmitoylcarnitine) were also tested in the presence of sparker malate at 100 μM. The substrates on MitoPlate S-1 were first dissolved by incubating the plate with 30 μL of Assay Mix, consisting of 2x BMAS, 6x Redox Dye MC and saponin (30 μg/mL) necessary for cell permeabilization in a 5 % CO_2_ incubator at 37 °C for 1 h before inoculating 40,000 cells per well in a volume of 30 μL. The reading of the signals was immediately carried out as described above and the results from the three technical replicates were averaged (Zhang et al., 2024). Substrate utilization was measured in real-time, and metabolic activity was quantified by calculating the Area Under the Curve (AUC) for total substrate utilization and the initial rate to determine the speed of substrate metabolism at the early stages of the experiment.

### ATP Quantification Assay

Huh7 cells were treated with vehicle or SC18 (30 μM) for 2 or 24 h, followed by treatment with oligomycin (10 μg/mL) or 2-deoxy-D-glucose (2-DG, 50 mM) for an additional 2 h. ATP levels were quantified using a luminescence-based ATP assay kit (Invitrogen, A22066). Lysate was mixed with standard reaction solution, containing luciferase, D-luciferin, reaction buffer, and DTT, in a white-walled 96-well plate. A standard curve was generated with 1 nM-1 µM ATP solutions. Background luminescence was subtracted, and data normalized to protein concentration. Seven experimental conditions were established: untreated cells (Vehicle), vehicle cells treated with oligomycin (Vehicle + Oligomycin), vehicle cells treated with 2-DG (Vehicle + 2-DG), SC18-treated cells (SC18–24h), SC18-treated cells with oligomycin (SC18–24h + Oligomycin), SC18-treated cells with 2-DG (SC18–24h + 2-DG) and SC18-treated cells (SC18–2h). ATP levels were measured in triplicate for each condition.

### L-Lactate Quantification Assay

Huh7 cells were treated with vehicle or SC18 (30 μM) for 2 & 24 h. L-Lactate levels were quantified using a colorimetric L-Lactate Assay Kit (Eton Bioscience, SKU 1200011002) according to the manufacturer’s protocol. Briefly, cell culture supernatants were mixed with L-Lactate assay solution. After incubation at 37°C for 30 min (without CO₂), the reaction was stopped by adding 0.5 M acetic acid per well. Absorbance was measured at 490 nm using a microplate reader. L-Lactate concentrations were determined using a standard curve generated from L-Lactate standards (0–3 mM) and calculated using linear regression analysis. Three experimental conditions were established: untreated cells (Vehicle), SC18-treated cells for 2 h (SC18–2h), and SC18-treated cells for 24 h (SC18–24h). L-Lactate levels were measured in triplicate for each condition.

### Oxygen Consumption Rate

An Agilent Seahorse XF Pro Analyzer (Agilent Technologies, Santa Clara, CA) was used to assess bioenergetic phenotypes of Huh7 cells in control, and SC18-treated for 2 h and 24 h. Huh7 cells were plated directly into the Agilent Seahorse XF Pro M cell culture microplates for 24 h, after which the media was changed to Seahorse XF assay medium containing no bicarbonate. The following compounds were used sequentially: Oligomycin A, an inhibitor of ATP synthase (energy production), followed by FCCP, an ionophore used as an uncoupling agent to assess maximal respiration. This was followed by Rotenone and Antimycin A, which inhibit Complex I and Complex III, respectively, causing disruption of the electron transport chain. Each cycle consisted of a 2-minute mixing period and a 3-minute measurement time. Three basal cycles were measured before compound injections, followed by three cycles after each injection. Each condition was tested in 12 replicate wells of three independent experiments to ensure statistical robustness.

### Statistics

After removing all the contaminants, only proteins with at least two peptides were included in the final dataset. Protein abundances were normalized by the total protein abundance in each sample. Then for each protein log2-transformed fold-changes were calculated as a log2-ratio of the intensity to the mean of all control replicates. Data were processed by Excel and R. Two-tailed Student's *t-tests* were used for statistical analysis between two groups, and ANOVA was used for statistical analysis between multiple groups. All statistical tests were two-sided and *P*-values less than 0.05 were considered statistically significant. Significance levels are displayed as *P<0.05, **P<0.01, and ***P<0.001.

## Funding and Support

This work and contributors at MUSC (DMT, ZY, JZ, LZ, EM) were supported by the South Carolina COBRE in Oxidants, Redox Balance and Stress Signaling (National Institutes of Health 5P20GM103542).

## Author contributions

**Seyed Majed Modaresi**: Writing – original draft, Visualization, Formal analysis, Data curation, Conceptualization. **Leilei Zhang**: Writing – original draft, Visualization, Formal analysis, Data curation. **Amir Ata Saei**: Writing – review & editing, Visualization, Formal analysis, Data curation. **Morris Degen**: Writing – review & editing, Visualization, Formal analysis, Data curation. **Mohammad Khavani**: Writing – review & editing, Visualization, Formal analysis, Data curation. **Hassan Gharibi**: Writing – review & editing, Visualization, Formal analysis, Data curation. **Ákos Végvári**: Writing – review & editing, Visualization, Formal analysis, Data curation. **Zhiwei Ye**: Writing – review & editing, Validation, Formal analysis, Data curation. **Jie Zhang**: Writing – review & editing, Visualization, Formal analysis, Data curation. **Evgeny Pavlov**: Writing – review & editing, Visualization, Formal analysis, Data curation. **Elizabeth A. Jonas**: Writing – review & editing, Visualization, Formal analysis, Data curation. **Kenneth D. Tew**: Writing – review & editing, Supervision, Funding acquisition, Formal analysis. **Sebastian Hiller**: Writing – original draft, Visualization, Supervision, Funding acquisition, Formal analysis, Data curation, Conceptualization. **Danyelle M. Townsend**: Writing – original draft, Visualization, Supervision, Funding acquisition, Formal analysis, Data curation, Conceptualization. **Eduardo N. Maldonado**: Writing – original draft, Visualization, Supervision, Funding acquisition, Formal analysis, Data curation, Conceptualization.

## Declaration of Competing Interests

The authors declare that they have no known competing financial interests or personal relationships that could have appeared to influence the work reported in this paper.

## Data Availability

All data is available upon request from the corresponding author. The mass spectrometry proteomics data have been deposited to the ProteomeXchange Consortium via the PRIDE partner repository with the dataset identifier PXD064542 (Data S1) (Vizcaino et al., 2014). The extracted protein abundances data and relevant outputs of data analysis are provided in Table S1. The cryo-EM density map has been deposited in the Electron Microscopy Data Bank (EMDB) under accession number EMD-71616. The corresponding atomic model has been deposited in the Protein Data Bank (PDB) under accession code 9PFZ (extended ID: PDB_00009PFZ).

## Footnotes

Appendix A Supplementary data associated with this article can be found in the online version at doi:10.1016/j.mocell.2026.100369.

## Footnotes

1 DOI: 10.1016/j.bpj.2015.08.015
