# Supplementary material for: Small molecule inhibition of voltage dependent anion channel 1 reroutes mitochondrial metabolite flux
Source: Mol Cells. 2026 May 13;49(7):100369. doi: 10.1016/j.mocell.2026.100369 (PMC13662758; doi:10.1016/j.mocell.2026.100369)
Supplement: Supplementary file 2 [file mmc2.docx]

**Supplementary Figure 1.**  **Biochemical preparation of VDAC1, membrane scaffold protein (MSP), and reconstitution of VDAC1 into lipid nanodiscs. (A)** Schematic of the construct used for recombinant expression of VDAC1, showing the expression vector (pET-21a) and the starting amino acid sequence of the protein product. **(B)** Ion exchange chromatography (IEX) profile of recombinantly expressed and refolded VDAC1. The monodisperse peak obtained during the first buffer-B gradient elution corresponds to properly refolded VDAC1 in detergent micelles. **(C)** Sodium Dodecyl Sulfate Polyacrylamide Gel Electrophoresis (SDS-PAGE) analysis of IEX-purified VDAC1. **(D)** Schematic of the construct used for recombinant expression of Membrane Scaffold Protein (MSP), showing the expression vector (pET-28a) and the starting amino acid sequence of the protein product. **(E)** SDS-PAGE analysis of MSP after immobilized metal affinity chromatography (IMAC). **(F)** SDS-PAGE analysis of MSP after TEV protease cleavage, before and after size exclusion chromatography (SEC). **(G)** SDS-PAGE analysis of DMPC (14:0) lipid-assembled MSP1D1 nanodiscs without VDAC1. **(H)** SEC profile comparing VDAC1-reconstituted nanodiscs with MSP1D1 nanodiscs assembled with and without VDAC1, demonstrating VDAC1 incorporation by shifts in hydrodynamic radius, dispersion, therefore the elution time. **(I)** SDS-PAGE analysis of VDAC1-reconstituted MSP1D1 nanodiscs, showing distinct bands corresponding to MSP and VDAC1 at the expected molecular weights. **(J)** Mass photometry profile of empty MSP1D1 nanodiscs assembled with DMPC (14:0), with a Gaussian fit indicating a molecular mass of 140 kDa. **(K)** Mass photometry profile of VDAC1-reconstituted MSP1D1 nanodiscs, with a Gaussian fit indicating a molecular mass of 167 kDa, Notably, unlike SDS-PAGE analysis, mass photometry accounts for the contribution of lipid molecules to the total measured mass.

**Supplementary Figure 2. TROSY spectra of [¹⁵N, ¹H]-labeled VDAC1 (E73V) under various experimental conditions.** Samples of 250 μM VDAC1 (E73V) were prepared and divided into two halves, with experiments conducted and analyzed in parallel to control for sample variability. **(A)** TROSY spectrum of apo VDAC1 (Sample 1). **(B)** TROSY spectrum of Sample 1 in the presence of 3 mM SC18 dissolved in DMSO-d₆. **(C)** TROSY spectrum of Sample 1 in the presence of 6 mM SC18 in DMSO-d₆. **(D)** TROSY spectrum of apo VDAC1 (Sample 2). **(E)** TROSY spectrum of Sample 2 treated with an equivalent volume of DMSO-d₆ (vehicle control) corresponding to the solvent volume used for the 6 mM SC18 condition. **(F)** Overlay of VDAC1 spectra comparing SC18 treatment (blue) and vehicle control (red), highlighting chemical shift perturbations induced by ligand binding.

**Supplementary Figure 3. AlphaFold3 co-folding predictions, MD simulation of VDAC1–SC18–NADH, and molecular dynamics based energetic characterization of ligand binding. (A–B)** Top-view Cα wire-frame projections comparing the predicted binding geometries of SC18 and NADH within the VDAC1 barrel. **(A)** AlphaFold3 co-folding prediction places SC18 (blue) and NADH (green) at distinct but proximal sites within the barrel lumen, with heteroatoms highlighted in red. **(B)** Equivalent projection of the NOE-based structural model of hVDAC-1 bound to β-NADH in detergent micelles (PDB: 6TIR), showing the experimentally restrained NADH binding position as determined by intermolecular NOE distance constraints. **(C)** Top-view overlay of the 20-conformer hVDAC1 NMR ensemble (PDB: 6TIQ/R; red ribbon traces) with the NOE-based NADH position (PDB: 6TIR; green sticks) aligned into the same frame. The spread of the individual conformers illustrates the conformational heterogeneity of the N-terminal helix and inter-strand loops sampled across the ensemble, while the β-barrel strands remain structurally converged. NADH localises to the barrel interior, contacting residues across multiple β-strands. **(D)** Molecular surface representation of the same complex. **(E–F)** AlphaFold3 confidence metrics across all three VDAC isoforms (VDAC1, circles; VDAC2, squares; VDAC3, triangles) for SC18-only, NADH-only, and SC18+NADH co-folding conditions. (C) Interface predicted TM-score (ipTM) and **(F)** overall ranking score are both highest for SC18 alone (mean ipTM = 0.94, mean ranking score = 0.93, blue shading), consistently across all three isoforms, indicating that SC18 engages VDAC with greater predicted structural confidence than NADH or the co-bound condition. **(G)** Representative snapshot from all-atom molecular dynamics simulation showing VDAC1 (red β-barrel) embedded in a hydrated lipid bilayer. Phospholipid acyl chains are shown in gold, with headgroup atoms visible at the bilayer interfaces. **(H)** Close-up of the co-binding pose from the MD trajectory showing SC18 and NADH occupying adjacent but distinct sub-pockets within the VDAC1 interior, with both ligands making extensive contacts with the barrel wall. **(I)** Pore radius profiles along the VDAC1 channel axis calculated by HOLE for Apo (gray), SC18 (purple dashed), NADH (green dashed), and SC18+NADH (red) conditions. The membrane-spanning region is highlighted. The inset shows that SC18+NADH reduces the minimum pore radius to 3.8 Å compared to 5.0 Å in the Apo state, consistent with partial occlusion of the conducting pathway and with the channel closure observed electrophysiologically. **(J)** MD-derived qualitative energetic analysis of ligand binding. (Left) MM-GBSA binding free energies (ΔG~bind~, qualitative) show that SC18+NADH yields the most favorable binding (–53.1 kcal/mol) relative to SC18 alone (–22.1 kcal/mol), suggesting energetic synergy in the co-bound state. (Centre left) Solvent-accessible surface area (SASA) decreases upon ligand binding, suggesting burial of hydrophobic surface. (Centre right) Total protein–ligand contacts increase substantially from SC18 alone (169) to NADH alone (681) to the co-bound state (1031), reflecting a more extensive binding interface when both ligands are present. (Right) Decomposition of interaction energies into electrostatic (ELE) and van der Waals (vdW) components indicates that both terms contribute to ligand binding within the hydrophilic VDAC1 lumen to qualitatively assessed different levels.

**Supplementary Figure 4. Biophysical characterization of VDAC1 in lipid bilayer nanodiscs. (A)** DSF measurements were performed on non-assembled MSP, assembled MSP1D1, and VDAC1-containing MSP1D1 nanodiscs with two different lipid compositions. Assembly of MSP with lipids significantly increases the melting temperature, while incorporation of the membrane protein VDAC1 into nanodiscs results in a biphasic melting temperature curve, with the melting temperature influenced by the lipid composition. **(B)** ^15^N- TRACT experiment done on the backbone of [^1^H, ^15^N] uniformly labelled VDAC1 reconstituted in LDAO detergent micelles and **(C)** MSP1D1 DMPC (14:0) nanodiscs, identifying a tumbling time of 20 and 25 ns, respectively.

**Supplementary Figure 5. Cryo-EM data collection, processing, and structural validation of VDAC1 reconstituted in MSP1D1 nanodiscs with DMPC (14:0) lipids. (A)** Representative negatively stained electron micrograph showing VDAC1–nanodisc particles distributed across the grid. Scale bar is 100 nm. **(B)** Selected 2D class averages of a cryo-electron microscopy dataset from iterative classification of 373,906 curated particles. Classes are ranked by particle count (highest top left) and labelled with their estimated resolution. The characteristic double-belt density flanking a central protein density corresponds to the MSP1D1 scaffold protein encircling human VDAC1 within a lipid bilayer patch. The diversity of orientations — including top views revealing the β-barrel lumen and tilted side views resolving the nanodisc belt — reflects good angular sampling of the dataset. **(C)** Data processing workflow in cryoSPARC. Beginning from 6,350 micrographs, automated blob picking yielded 8,260,593 candidate particles. Following exposure curation (7,550,837 particles) and particle picking and extraction (6,668,471 particles), iterative 2D classification reduced the dataset to 373,906 high-quality particles, which were used for ab initio 3D reconstruction yielding three initial classes). **(D)** Refinement pipeline. The selected particle stack was subjected to non-uniform (NU) refinement, yielding an initial map at 6.2 Å. Heterogeneous refinement separated conformational heterogeneity (7.9 Å). Subsequent iterative rounds of ab initio reconstruction, heterogeneous refinement, and NU-refinement progressively improved the map from 6.8 Å to a final reconstruction at 5.4 Å. Teal density maps are shown at each stage. **(E)** Quality metrics of the final reconstruction. (Left) 2D angular distribution plot displaying the elevation and azimuth of particle orientations contributing to the final map; warm colors indicate preferred orientations. (Right) Gold-standard Fourier Shell Correlation (GSFSC) curve indicating a nominal resolution of 5.39 Å (0.143 criterion) after masking correction; curves for no-mask (6.7 Å), loose mask (5.9 Å), tight mask (5.3 Å), and phase-randomisation-corrected mask (5.4 Å) are shown. **(F)** Structural models of human VDAC1 used for comparative analysis, shown as ribbon representations (black) with secondary structural elements visible. From left to right: X-ray crystal structures of human VDAC1 (PDB: 2JK4) and mouse VDAC1 (PDB: 3EMN), AlphaFold2 structure prediction of human VDAC1 (UniProt: P21796), and solution NMR structure of human VDAC1 (E73V) in LDAO detergent (PDB: 6TIQ). Species and experimental context are indicated by icons. All models share the conserved 19-stranded β-barrel architecture with a central N-terminal α-helix, though minor variations in helix positioning are apparent across structures, likely reflecting differences in the experimental or computational environment.

**Supplemental Figure 6. Saturation Transfer Difference (STD) NMR spectroscopy demonstrates SC18 binding to VDAC1 in lipid nanodiscs. (A)** ¹H STD NMR spectra of SC18 in the presence of three conditions: empty MSP1D1 nanodiscs without VDAC1 (blue, negative control), VDAC1 reconstituted in LDAO detergent micelles (orange), and VDAC1 reconstituted in MSP1D1 nanodiscs (green). For each condition, off-resonance (I_off_), on-resonance (I_on_), and difference (STD = I_off_ − I_on_) spectra are shown. Shaded regions denote spectral windows used for quantification: aromatic protons (6.4–8.9 ppm, SC18-specific, purple), olefinic/OCH₂ protons (4.6–6.5 ppm, green), and aliphatic protons (0.2–4.5 ppm, orange). Prominent LDAO CH₂/CH₃ signals are indicated in the LDAO condition. **(B)** Expanded STD difference spectra of the aromatic region (6.5–9.0 ppm), where only isolated SC18 resonance contributes to the signal. The negative control (empty nanodiscs) yields a peak SNR of −3, consistent with noise. VDAC1 in LDAO micelles produces no resolvable STD signal at 0.5 mM ligand concentration (SNR = 2.1), while VDAC1 in MSP1D1 nanodiscs yields a clear positive STD response (peak SNR = +8), indicating apparent binding of SC18 to VDAC1 in the lipid bilayer environment. The grey band denotes the ±3σ noise envelope. Higher engagement observed in lipid bilayers likely reflects the partitioning of the hydrophobic SC18 into LDAO micelles, which sequesters the compound and reduces its effective free concentration, thereby suppressing detectable VDAC1 interaction in the low micromolar concentration regime employed here. **(C)** Reference ¹H NMR spectrum of SC18 in DMSO-d6, confirming the tentative chemical shift assignments of the aromatic (6–9 ppm, inset) proton resonances used to identify SC18-specific STD signals. **(D)** Quantification of positive STD integrals (noise-normalised, σ = 1) in the SC18-specific aromatic region (6.4–8.9 ppm, top) and the aliphatic region (0.5–4.5 ppm, bottom, which additionally includes contributions from DMPC and LDAO alkyl groups). Aromatic STD integrals reveal a significant signal only in the nanodisc condition (+VDAC MSP1D1 = 3.98), which markedly exceeds both the negative control (−VDAC = 0.96) and the LDAO condition (+VDAC LDAO = 2.08), the latter remaining indistinguishable from background (*** p < 0.001, block-permutation pairwise test). The aliphatic integrals follow the same trend (4.3, 12.3, 36.0), though the larger values in the nanodisc condition additionally reflect lipid resonance contributions from DMPC. The grey dashed line indicates the 95th percentile of the noise-floor integral null distribution.
